# A mixture of extracts from natural ingredients reduces the neurotoxic polarization of microglia via modulating NF‐κB/NF‐E2‐related factor 2 activation

**DOI:** 10.1002/fsn3.4045

**Published:** 2024-02-20

**Authors:** Shuge Gui, Junjun Ni, Shinsuke Mizutani, Norihiro Shigematsu, Hiroshi Nakanishi, Haruhiko Kashiwazaki, Zhou Wu

**Affiliations:** ^1^ Department of Oral and Maxillofacial Surgery, Faculty of Dental Science Kyushu University Fukuoka Japan; ^2^ Key Laboratory of Molecular Medicine and Biotherapy, School of Life Science Beijing Institute of Technology Beijing China; ^3^ Section of Geriatric Dentistry and Perioperative Medicine in Dentistry, Division of Maxillofacial Diagnostic and Surgical Sciences, Faculty of Dental Science Kyushu University Fukuoka Japan; ^4^ Yamada Institute for Health Science, R & D Department Yamada Bee Company, Inc. Okayama Japan; ^5^ Department of Pharmacology, Faculty of Pharmacy Yasuda Women's University Hiroshima Japan; ^6^ Department of Aging Science and Pharmacology, Faculty of Dental Science Kyushu University Fukuoka Japan; ^7^ OBT Research Center, Faculty of Dental Science Kyushu University Fukuoka Japan

**Keywords:** microglial polarization, natural ingredients, NF‐E2‐related factor 2, NF‐κB

## Abstract

Neurotoxic microglia‐provoked neuroinflammation is implicated in cognitive decline in Alzheimer's disease (AD). Supplementation with *Ginkgo biloba*, phosphatidylserine, *Curcuma longa*, and propolis is reported to improve the cognitive functions of elderly people; however, the underlying mechanisms of this combination of natural ingredients are unknown. We investigated the effects of a mixture of extracts from propolis, *Coffea arabica*, *Gotu kola*, phosphatidylserine, *Ginkgo biloba*, and *Curcuma longa* (mixture) on microglia polarization after exposure to amyloid β_1‐42_ (Aβ_1‐42_, 1 μM) and lipopolysaccharide from *Porphyromonas gingivalis* (PgLPS, 1 μg/mL), using MG6 and BV2 microglial cells. Exposure to Aβ_1‐42_ and PgLPS (AL) raised the mRNA expression of IL‐1β, TNF‐α, and IL‐6, nuclear translocation of p65 NF‐κB in MG6 cells and BV2 cells, and mitochondrial reactive oxygen species (ROS) production in MG6 cells. The mixture dramatically suppressed the mRNA expression of IL‐1β, TNF‐α, and IL‐6, but significantly promoted that of IL‐10, TGFβ1, and BDNF in AL‐exposed MG6 and BV2 cells. Furthermore, the mixture significantly suppressed the nuclear translocation of p65 NF‐κB but significantly promoted that of NF‐E2‐related factor 2 (Nrf2) in AL‐exposed MG6 and BV2 cells. Furthermore, the mixture significantly ameliorated mitochondrial ROS production but increased mitochondrial membrane potential in MG6 cells. These observations strongly suggest that the mixture demotes the neuropathic polarization of microglia by modulating NF‐κB/Nrf2 activation and improving mitochondrial functions. This study supplies the potential mechanisms of the efficacy of a combination of natural ingredients that can be applied in the prevention of cognitive decline in AD and aging by targeting microglia‐mediated neuroinflammation.

## Graphical abstract

The present study found that the mixture of natural ingredients reduced neurotoxic polarization of microglia via modulating NF‐κB/Nrf2 activation and alleviating mitochondrial damage during exposure to amyloid β and TLR ligands. The explored potential mechanism of natural ingredient combination will contribute to generating functional foods for prevention and early intervention of cognitive decline in dementia as well as aging.

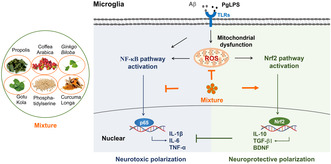

## 1 INTRODUCTION

Neuroinflammation is implied in the initiation and pathological process of dementia, such as Alzheimer's disease (AD) (Heneka et al., 2015; Tran et al., 2022), and microglia play critical roles in neuroinflammation (Salter & Stevens, 2017; Wu, Sun, et al., 2013; Wu, Zhu, et al., 2013). As highly plastic cells, microglia can be derived into neurotoxic or neuroprotective phenotypes when exposed to stimuli. Neurotoxic microglia exacerbate neuroinflammation by producing proinflammatory effectors, such as TNF‐α, IL‐1β, and IL‐6, resulting in neuronal damages (Jiang et al., 2021; Mirabella et al., 2021; Wu, Sun, et al., 2013; Wu, Zhu, et al., 2013), while neuroprotective microglia ameliorate neuroinflammation by producing anti‐inflammatory and neurotrophic effectors, including IL‐10, TGFβ1, and BDNF, resulting in neuronal benefits (Parkhurst et al., 2013; Wu et al., 2005). As a characteristic hallmark of AD, amyloid (A) β aggregation starts 15–20 years before the clinical symptoms become apparent in the brain of AD patients, which is believed to be the initial event of the pathological process (Randall et al., 2012). Aβ_1‐42_ has been shown to drive microglia into neurotoxic (pro‐inflammatory) phenotypes depending on their form and concentration (Quiroga et al., 2022; Yang et al., 2021). Toll‐like receptors (TLRs) on microglia sense invading pathogens and endogenous danger molecules; among these, TLR2 and TLR4 on microglia trigger proinflammatory responses (Fellner et al., 2013; Jana et al., 2008). Previously, lipopolysaccharide from *Porphyromonas gingivalis* (PgLPS), the ligand of TLR2 and TLR4, was found in the autopsy brains of AD patients (Poole et al., 2013). PgLPS is reported to drive microglia into proinflammatory phenotypes through TLR2 and TLR4 (Liu et al., 2013; Tran et al., 2022; Wu et al., 2017).

NF‐κB is known as a master transcription factor for amplifying inflammation (Liu et al., 2017), which pivotally mediates the proinflammatory responses of microglia (Jin, Liu, Zhang, Zhong, Du, Qian, et al., 2019; Jin, Liu, Zhang, Zhong, Du, Qian, Yao, et al., 2019). On the other hand, nuclear factor E2‐related factor 2 (Nrf2) is recognized as a transcription factor for attenuating inflammation, which promotes anti‐inflammatory responses in microglia (Cui et al., 2021; Okorji et al., 2016). Additionally, crosstalk between NF‐κB and Nrf2 is considered to influence the course of inflammation (Wardyn et al., 2015).

Previous studies have shown that natural ingredients can prevent memory decline, and that this is partly dependent on their anti‐inflammatory and antioxidant efficacies (Delerue et al., 2021; Jin, Liu, Zhang, Zhong, Du, Qian, et al., 2019; Jin, Liu, Zhang, Zhong, Du, Qian, Yao, et al., 2019; Zhu et al., 2018). *Coffea arabica* (CA), the major component of coffee, has been found to reduce inflammation and protect neurons (Islam et al., 2018). Phosphatidylserine (PS), a component in soybeans (Richter et al., 2013), has been shown to attenuate neuroinflammation (De Simone et al., 2003; Huynh et al., 2002). *Curcuma longa* (CL), a component in curry spices, is known to reduce proinflammatory mediator production by cells, including microglia (Shi et al., 2015; Sowndhararajan et al., 2018; Zhang et al., 2022). PS, *Ginkgo biloba* (GB), and *Gotu kola* (GK) have been reported to improve the memory functions in individuals (Kandiah et al., 2021; Ma et al., 2022; McDaniel et al., 2003; Puttarak et al., 2017), in addition to their effects of reducing the production of proinflammatory effectors by cells, including microglia (Shi et al., 2015; Sowndhararajan et al., 2018; Zhang et al., 2022), Furthermore, propolis, a resinous mixture that honeybees produce by mixing saliva and beeswax, has been determined to prevent memory decline in humans by its anti‐inflammatory and neuroprotective effects (Ni et al., 2017; Wu, Sun, et al., 2013; Wu, Zhu, et al., 2013; Zhu et al., 2018). Although the benefits of CA, GB, PS, GK, CL, and propolis in humans have been detected, no evidence has been reported on the synergistic effects of those ingredients on neuroinflammation.

Recent clinical research has shown that taking supplements containing GB, PS, propolis, and curcumin (a major polyphenolic compound of CL) for 12 weeks improves the memory functions of humans without any side effects (Takashi et al., 2020). However, the molecular mechanism underlying the combined effects is unclear. In this study, we tested our hypothesis that a mixture of propolis, CA, GK, PS, GB, and CL (mixture) would synergistically dampen microglia‐mediated neuroinflammation. We explored the molecular mechanisms of the mixture in regulating microglial phenotype polarization during exposure to soluble Aβ_1‐42_ and PgLPS (AL).

## 2 MATERIALS AND METHODS

### 2.1 Materials

The c‐myc‐immortalized mouse microglial cell line MG6 (Cat: RCB2403) was purchased from RIKEN Cell Bank; BV2 cells (the immortalized mouse microglial cell line) were obtained from ACCEGEN (Cat: ABC‐TC212S). Eagle's minimum essential medium and Dulbecco's modified Eagle's medium were purchased from NISSUI PHARMACEUTICAL CO., LTD; Fetal Bovine Serum (FBS), Penicillin–Streptomycin, Hank's Balanced Salt Solution (HBSS), and L‐Glutamine were purchased from Gibco Invitrogen; β‐mercaptoethanol and insulin were bought from Sigma. Aβ_1‐42_ (Cat:4214‐v) was obtained from Peptide Institute; lipopolysaccharide from Porphyromonas gingivalis (Cat:tlrl‐pglps) was purchased from InvivoGen; Brazilian green propolis ethanol extract power (LY3‐004, standardized to contain 8.0% artepillin C and 0.14% culifolin, propolis), *Coffea arabica* (CA) (09994459 N995), *Gotu kola* (GK) (GK171216), *Ginkgo biloba* (GB) (GB20190110), phosphatidylserine (PS) (18B0268), and *Curcuma longa* (CL) (AI/TE/10151118) were obtained from Yamada Bee Company Inc. Cell‐Counting Kit‐8 (CCK‐8) and the JC‐1 MitoMP Detection Kit were purchased from Dojindo Molecular Technologies. RNAiso Plus was obtained from Takara; ReverTra Ace® qPCR RT Master Mix and THUNDERBIRD® SYBR® qPCR Mix were purchased from TOYOBO. Western blot antibodies: mouse anti‐phospho‐IκBα, rabbit anti‐IκBα were obtained from Cell Signaling Technology; rabbit anti‐NF‐κB p65, mouse anti‐β Actin antibodies were obtained from Abcam; mouse anti‐Lamin B1 antibody was purchased from Proteintech and mouse anti‐NRF2 antibody was purchased from MEDICAL BIOLOGICAL LABORATORIES CO; Amersham ECL Horseradish peroxidase anti‐mouse IgG and anti‐rabbit IgG were purchased from GE Healthcare; ImmunoStar LD was purchased from Fujifilm; Nuclear Extraction Kit was obtained from Abcam. The mouse TNF‐alpha Quantikine ELISA Kit was obtained from R&D Systems, Inc; MitoSOX Red was obtained from Thermo Fisher SCIENTIFIC. Paraformaldehyde (PFA) and Triton™ X‐100 were obtained from Sigma Aldrich; Bovine Serum Albumin (BSA) was obtained from Wako; Alexa Fluor® 488 AffiniPure Donkey Anti‐Rabbit IgG and Donkey Anti‐Mouse IgG were purchased from Jackson ImmunoResearch Laboratories Inc; Hoechst was obtained from Sigma‐Aldrich. Vectashield anti‐fading medium was obtained from Vector Laboratories.

### 2.2 Microglial cell culture

MG6 cells were cultured in Dulbecco's modified Eagle's medium supplemented with 10% FBS, 1% Penicillin–Streptomycin, 100 μM β‐mercaptoethanol, and 10 μg/mL insulin in accordance with what was previously described (Wu, Sun, et al., 2013; Wu, Zhu, et al., 2013). MG6 cells were treated with Aβ_1‐42_ (1 μM) or pretreated with Aβ_1‐42_ 1 h before being treated with PgLPS (1 μg/mL). BV2 cells were cultured in Eagle's minimum essential medium supplemented with 10% FBS and 1% Penicillin–Streptomycin at 37°C and 5% CO_2_ in a humidified atmosphere. BV2 cells were pretreated with Aβ_1‐42_ 1 h before being treated with PgLPS (1 μg/mL).

### 2.3 Cell viability assays

MG6 cells and BV2 cells were seeded in 96‐well plates that were incubated with propolis, CA, GK, GB, PS, or CL alone or a mixture of propolis, CA, GK, GB, PS, and CL together (mixture) for 24 h, 48 h, and 72 h. MG6 cells and BV2 cells were incubated with soluble Aβ_1‐42_ (1 μΜ) or Aβ_1‐42_ (1 μΜ) with PgLPS (1 μg/mL) for 24 h, 48 h, and 72 h. At different time points, 10 μL of CCK‐8 solution was added to each well, and the plates were incubated for 1 h. Cell viability was measured with the Cell‐Counting Kit‐8 according to the manufacturer's protocol.

### 2.4 Real‐time PCR analysis

mRNA was isolated from MG6 cells and BV2 cells after being exposed to Aβ_1‐42_ or AL in the presence or absence of the mixture. Total mRNA was collected and extracted using RNAiso Plus according to the manufacturer's protocol. And 1 μg of extracted mRNA was reverse transcribed to cDNA using a ReverTra Ace® qPCR RT Master Mix. The cDNA was amplified in duplicate using THUNDERBIRD® SYBR® qPCR Mix with a StepOnePlus™ Real‐Time PCR System. The sequences of the primer pairs are shown in Table S1. The level of gene expression was normalized by internal control β‐actin, and data were evaluated by 2^−ΔΔCT^.

### 2.5 Western blot analysis

MG6 cells were harvested after exposure to AL. The nuclei of MG6 cells were isolated by a nuclear extraction kit. In brief, samples were exposed to AL in the presence or absence of the mixture. The protein samples were separated using 8% or 10% SDS polyacrylamide gels and then transferred to nitrocellulose membranes. After blocking with 5% skim milk for 1 h at room temperature, the membranes were incubated with the primary antibodies of phospho‐IκBα, IκBα, NF‐κB p65, NRF2, β‐Actin, and Lamin B1 overnight at 4°C. After being washed, the membranes were incubated with HRP‐conjugated secondary antibodies (anti‐mouse IgG, anti‐rabbit IgG) for 2 h at room temperature. The bands were detected by ImmunoStar LD with an image analyzer (LAS‐4000 mini, JP).

### 2.6 Enzyme‐linked immunosorbent assay (ELISA)

Cultured MG6 cells (5 × 10^5^ cells/ml), which were exposed to Aβ_1‐42_ or AL in the presence or absence of the mixture, were incubated in 5% CO_2_ at 37°C. The supernatants were collected at 3, 6, 12, and 24 h after the above exposure. The released TNF‐α from microglia was determined by the ELISA Kit according to the manufacturer's protocol.

### 2.7 Immunofluorescence imaging

For immunofluorescence staining of MG6 cells and BV2 cells, the cells were fixed with a 4% PFA solution for 10 min and permeabilized with 0.1% Triton™ X‐100 for 5 min at room temperature. After being incubated with 3% BSA for 1 h, the cells were treated with NF‐κB p65 (1:5000) or NRF2 (1:1000) overnight at 4°C. After washing with PBS, the cells were applied with Alexa Fluor® 488 Anti‐Rabbit IgG (1:1000) or Anti‐Mouse IgG (1:1000) at room temperature for 2 h. After washing with PBS, cell nuclei were counterstained with Hoechst (1:500) for 5 min and mounted in Vectashield anti‐fading medium. Fluorescence images were captured by the CLMS (2si Confocal Laser Microscope, Nikon).

### 2.8 Fluorescence detection of mitochondrial ROS

Mitochondrial ROS production in MG6 microglia was determined using MitoSOX Red  (Wu, Zhu, et al., 2013). Briefly, MG6 microglia on 8‐well chamber slides (1 × 10^5^ cells/well) were exposed to Aβ_1‐42_ or AL in the presence or absence of the mixture. The cells were collected at 1 h after treatments and then incubated in HBSS containing 1 μM MitoSOX Red for 30 min at 5% CO_2_ at 37°C. After incubation, the cells were washed with warm HBSS and mounted in a warm buffer for imaging. Images were collected with a 40× objective lens using a confocal laser scanning microscope (C2si, Nikon).

### 2.9 JC‐1 fluorescence staining

The mitochondrial membrane potential of MG6 cells was assessed using JC‐1. Staining was conducted according to the manufacturer's protocols. Briefly, MG6 cells on 8‐well chamber slides (1 × 10^5^ cells/well) were exposed to Aβ_1‐42_ or AL in the presence or absence of the mixture. The cells were then incubated with 2 μM JC‐1 fluorescence dye at 37°C for 30 min and rinsed with HBSS. Images were collected with a 20× objective lens using a confocal laser scanning microscope (C2si, Nikon).

### 2.10 Statistical analysis

All data were generated by at least three replicates from independently prepared samples. The data are presented as the mean ± SD. The statistical analyses were performed using one‐way (ANOVA) with Tukey's post hoc test using the GraphPad Prism software package. *p* values of <.05 were considered to indicate statistical significance (GraphPad Software Inc., San Diego, CA, USA).

## 3 RESULTS

### 3.1 The effects of a mixture on microglial viability

**FIGURE 1:**
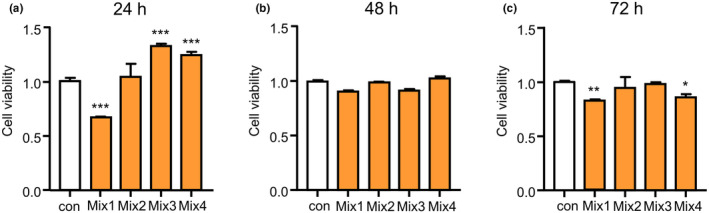
The effect of the natural ingredient mixture on microglial viability. (a) 24 h, (b) 48 h, and (c) 72 h after treatment with the natural ingredient mixture. Mixture 1: propolis (20 μg/mL) with CA (0.25 mg/mL), GK (100 μg/mL), PS (25 μg/mL), GB (6.25 μg/mL), and CL (0.25 μg/mL). Mixture 2: propolis (20 μg/mL) with CA (0.5 mg/mL), GK (200 μg/mL), PS (50 μg/mL), GB (12.5 μg/mL), and CL (0.5 μg/mL). Mixture 3: propolis (20 μg/mL) with CA (1 mg/mL), GK (400 μg/mL), PS (100 μg/mL), GB (25 μg/mL), and CL (1 μg/mL). Mixture 4: propolis (20 μg/mL) with CA (2 mg/mL), GK (800 μg/mL), PS (200 μg/mL), GB (50 μg/mL), and CL (2 μg/mL) on the cell viability of MG6 cells. Each column and bar represents the mean ± SD (*n* = 3, each). Asterisks indicate a statistically significant difference from the value in the control (con) group (*p < .05, **p < .01, ***p < .001, one‐way ANOVA).

We first performed screening to determine the suitable amounts of signal ingredients for microglial viability using CCK8. In comparison to control (no ingredient treatment) cells, the mean MG6 cell viability was not significantly altered until 72 h after treatment with ingredients at the following concentrations: up to 20 μg/mL propolis, up to 2 mg/mL CA, up to 800 μg/mL GK, up to 200 μg/mL PS, up to 50 μg/mL GB, and up to 2 μg/mL CL (Figures S1–S6). Based on the available concentration of signal ingredient, we next examined the suitable concentration of the mixture of propolis (20 μg/mL) with CA (0.25 mg/mL), GK (100 μg/mL), PS(25 μg/mL), GB (6.25 μg/mL), and CL (0.25 μg/mL) as mixture 1; CA (0.5 mg/mL), GK (200 μg/mL), PS (50 μg/mL), GB (12.5 μg/mL), and CL (0.5 μg/mL) as mixture 2; CA (1 mg/mL), GK (400 μg/mL), PS (100 μg/mL), GB (25 μg/mL), and CL(1 μg/mL) as mixture 3; and CA (2 mg/mL), GK (800 μg/mL), PS (200 μg/mL), GB (50 μg/mL), and CL (2 μg/mL) as mixture 4 on the cell viability of MG6 cells. In comparison to control cells, the mean MG6 cell viability was not significantly altered until 72 h after adding the mixture of propolis (20 μg/mL), CA (0.5 mg/mL), GK (200 μg/mL), PS (50 μg/mL), GB (12.5 μg/mL), and CL (0.5 μg/mL) (mixture 2; Figure 1a–c). The mean MG6 cell viability was significantly altered as follows: fallen at 24 h and 72 h after treatment with mixture 1, raised at 24 h but fallen at 48 h after treatment with mixture 3, and raised at 24 h but fallen at 72 h after adding mixture 4 (Figure 1a–c). Additionally, in comparison to control cells, the mean BV2 cell viability was not significantly altered until 72 h after adding mixture 2 (Figure S8). Therefore, we used mixture 2 for the subsequent experiments.

### 3.2 Generation of microglial polarization in vitro model by exposure to soluble Aβ and PgLPS

**FIGURE 2:**
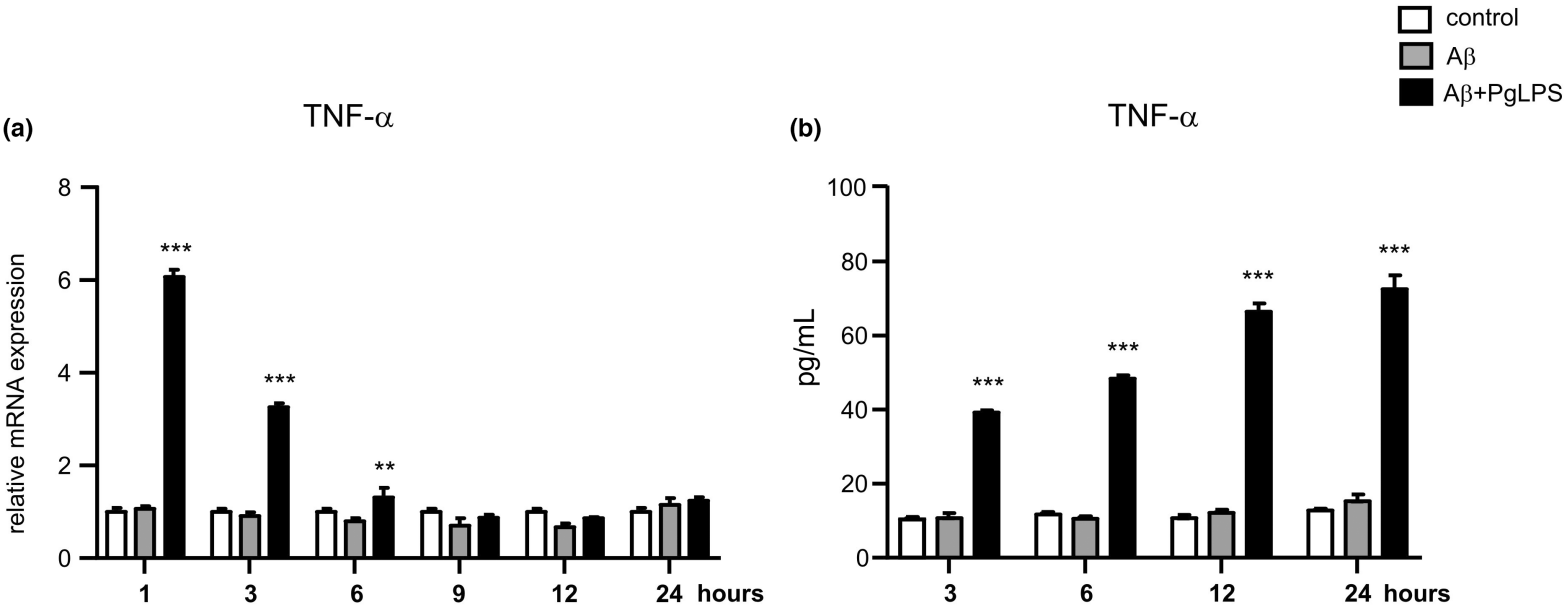
TNF‐α production in microglia during exposure to Aβ and PgLPS. (a) Time course of TNF‐α mRNA expression in MG6 cells after either exposure to Aβ (1 μM) or exposure to Aβ (1 μM) and PgLPS (1 μg/mL). Each column and bar represents the mean ± SD (*n* = 3, each). Asterisks indicate a statistically significant difference from the value in the control group (**p < .01, ***p < .001, one‐way ANOVA). (b) Time course of TNF‐α secretion by MG6 cells after exposure to either Aβ alone (1 μM) or Aβ (1 μM) and PgLPS (1 μg/mL). Each time point and bar represents the mean ± SD (*n* = 3, each). Asterisks indicate a statistically significant difference from the value in the control (con) group (**p < .01, ***p < .001, one‐way ANOVA).

To explore the effects of the mixture on microglial polarization in AD, we attempted to generate a stable in vitro model of microglia mimicking the brain environment before the onset of AD. MG6 cells were exposed to soluble Aβ (before the formation of plaques) and PgLPS (coligand of TLR2 and TLR4), which were found in the autopsy brains of AD patients (Poole et al., 2013). In comparison to unexposed MG6 cells (control), the mean cell viability was not significantly altered until 72 h after adding Aβ_1‐42_ (1 μM) or Aβ_1‐42_ (1 μM) and PgLPS (1 μg/mL) (AL, Figure S7). In comparison to control cells, TNF‐α mRNA expression was significantly induced and peaked at 1 h (6.1‐fold rise), lasted until 3 h (3.3‐fold rise) and 6 h (1.3‐fold rise) (Figure 2a), and TNF‐α amount in the culture medium of microglia was significantly raised from 3 h (4.5‐fold rise) until 24 h (7.9‐fold rise) after exposure to AL (Figure 2b). However, neither TNF‐α mRNA expression nor TNF‐α production and secretion were increased after exposure to Aβ_1‐42_ alone (Figure 2a, b). Thus, Aβ_1‐42_ and PgLPS‐exposed MG6 cells can be used as a stable in vitro model of microglial polarization mimicking microglia in the environment of the AD brain.

### 3.3 The mixture suppressed the expression of proinflammatory genes in microglia during exposure to soluble Aβ and PgLPS

**FIGURE 3:**
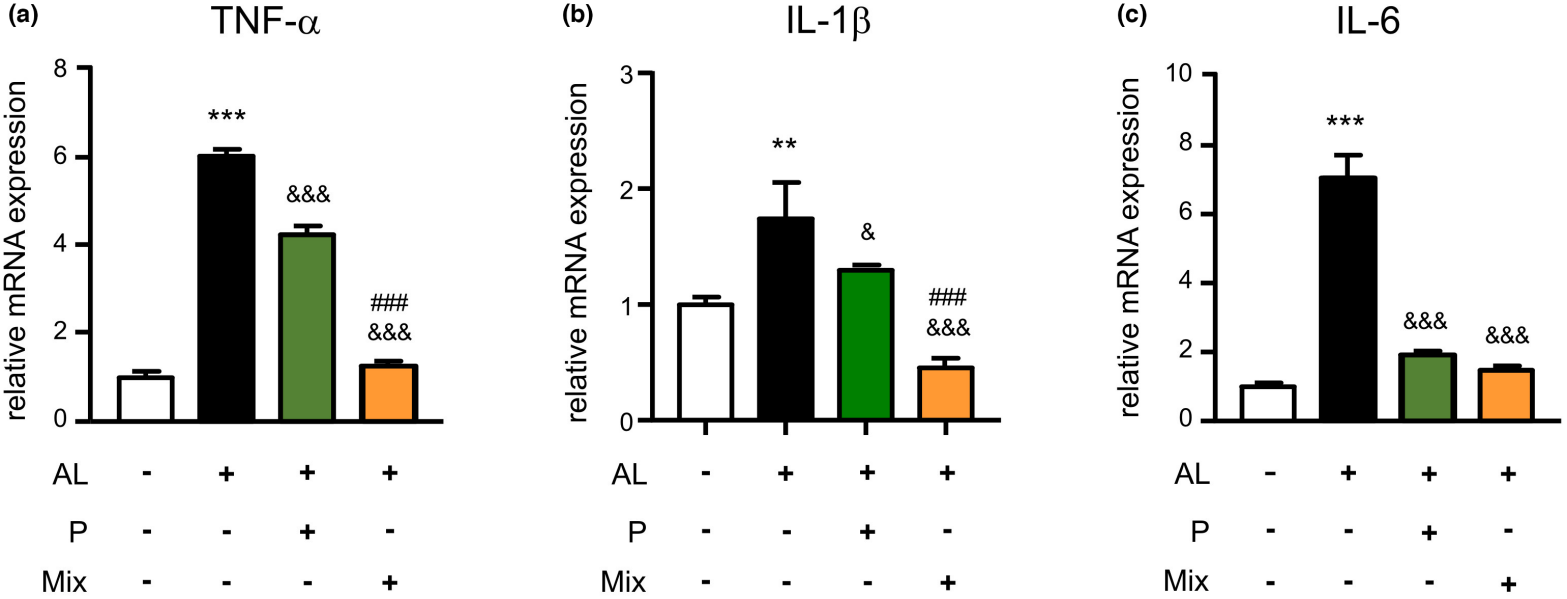
Mixture suppressed the expression of proinflammatory genes in microglia during exposure to Aβ and PgLPS. The mRNA expression of TNF‐α (a), IL‐1β (b), and IL‐6 (c) at 1 h after exposure to Aβ and PgLPS (AL) with or without pretreatment with propolis (P) or mixture (Mix). Each column and bar represents the mean ± SD (*n* = 3, each). Asterisks indicate a statistically significant difference from the value in the control group (**p < .01, ***p < .001, one‐way ANOVA). Swords indicate a statistically significant difference from the value in the Aβ and PgLPS‐exposed group (^&^ *p* < .05, ^&&^ *p* < .01, ^&&&^ *p* < .001, one‐way ANOVA). Hash marks indicate a statistically significant difference from the value in the propolis and mixture group (^###^ *p* < .001, one‐way ANOVA).

**FIGURE 4:**
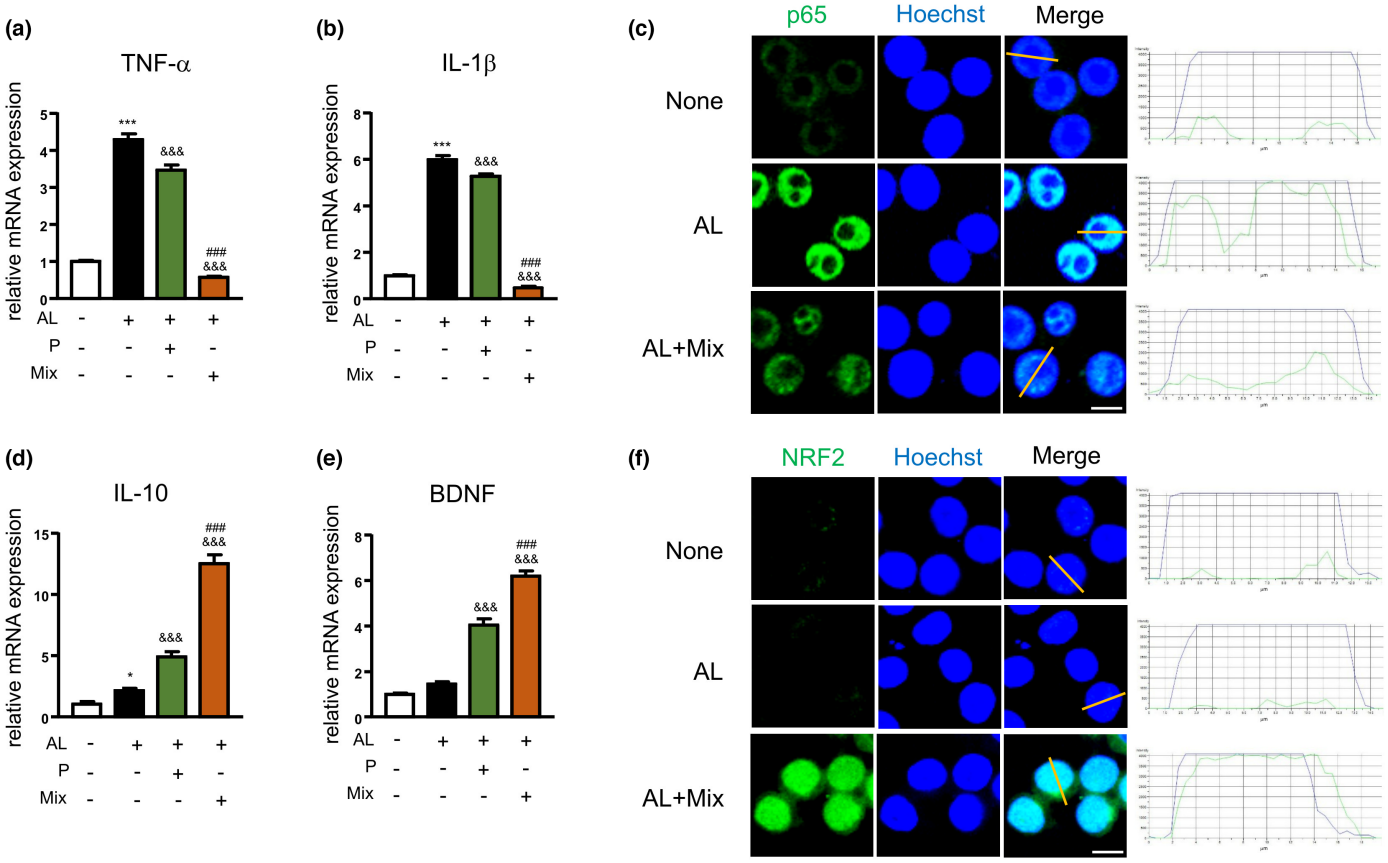
The mixture regulated the gene expression and NF‐κB/Nrf2 activation in BV2 microglia during exposure to Aβ and PgLPS. The mRNA expression of TNF‐α (a), IL‐1β (b) at 1 h after exposure to Aβ and PgLPS (AL) with or without pretreatment with propolis (P) or mixture (Mix). (c) Immunofluorescent CLMS images indicating the nuclear translocation of p65 (green) in BV2 cells with Hoechst‐stained nuclei (blue) after exposure to AL for 1 h. Scale bar, 10 μm. The mRNA expression of IL‐10 (d) and BDNF (e) at 1 h after exposure to AL with or without pretreatment with propolis (P) or mixture (Mix). (f) Immunofluorescent CLMS images indicating the nuclear translocation of Nrf2 (green) in BV2 cells with Hoechst‐stained nuclei (blue) after exposure to AL for 1 h. Scale bar, 10 μm. Each column and bar represents the mean ± SD (*n* = 3, each). Asterisks indicate a statistically significant difference from the value in the control group (*p < .05, ***p < .001, one‐way ANOVA). Swords indicate a statistically significant difference from the value in the Aβ and PgLPS‐exposed group (^&&&^ *p* < .001, one‐way ANOVA). Hash marks indicate a statistically significant difference from the value in the propolis and mixture group (^###^ *p* < .001, one‐way ANOVA).

Using our generated model, we investigated the effects of the mixture on proinflammatory gene expression in AL‐exposed microglia. As shown in Figure 3, the mRNA expression levels of TNF‐α, IL‐1β, and IL‐6 were raised in the AL‐exposed MG6 cells as soon as 1 h after exposure to AL in comparison to control MG6 cells (6.1‐fold, 1.7‐fold, and 7‐fold rises). In comparison to the AL‐exposed MG6 cells, propolis alone significantly mitigated the mRNA expression of TNF‐α, IL‐1β, and IL‐6 in the AL‐exposed MG6 cells at 1 h (29.6%, 25.5%, and 72.7% rises). The mixture significantly reduced the expression of TNF‐α, IL‐1β, and IL‐6 at 1 h in the AL‐exposed MG6 cells (79%, 73.9%, and 79.1% decreases). Notably, the mixture significantly reduced the expression of TNF‐α and IL‐1β (but not IL‐6) at 1 h compared with propolis alone in AL‐exposed MG6 cells (70%, and 64.9% decreases). The significant inhibitory effect of the mixture on the mRNA expression of TNF‐α, IL‐1β, and IL‐6 in MG6 cells lasted until 3 h after exposure to AL (data not shown). In comparison to the control BV2 cells, the mRNA expression of TNF‐α and IL‐1β was raised in the BV2 cells exposed to AL 1 h after exposure (4.3‐fold and 6‐fold rises) (Figure 4a,b). In comparison to the AL‐exposed BV2 cells, propolis alone significantly mitigated the mRNA expression of TNF‐α and IL‐1β in the AL‐exposed BV2 cells at 1 h (19.2% and 12.1% decrease). The mixture significantly reduced the expression of TNF‐α and IL‐1β in the AL‐exposed BV2 cells at 1 h (86.7% and 91.9% decreases). Furthermore, in comparison to propolis alone, the mixture significantly inhibited the mRNA expression of TNF‐α and IL‐1β in AL‐exposed BV2 cells at 1 h (83.5% and 90.8% decreases) (Figure 4a,b). These observations demonstrate that the mixture reduces the neurotoxic polarization of microglia during exposure to Aβ_1‐42_ and TLR ligands.

### 3.4 The mixture raised the expression of anti‐inflammatory genes in microglia during exposure to soluble Aβ and PgLPS

**FIGURE 5:**
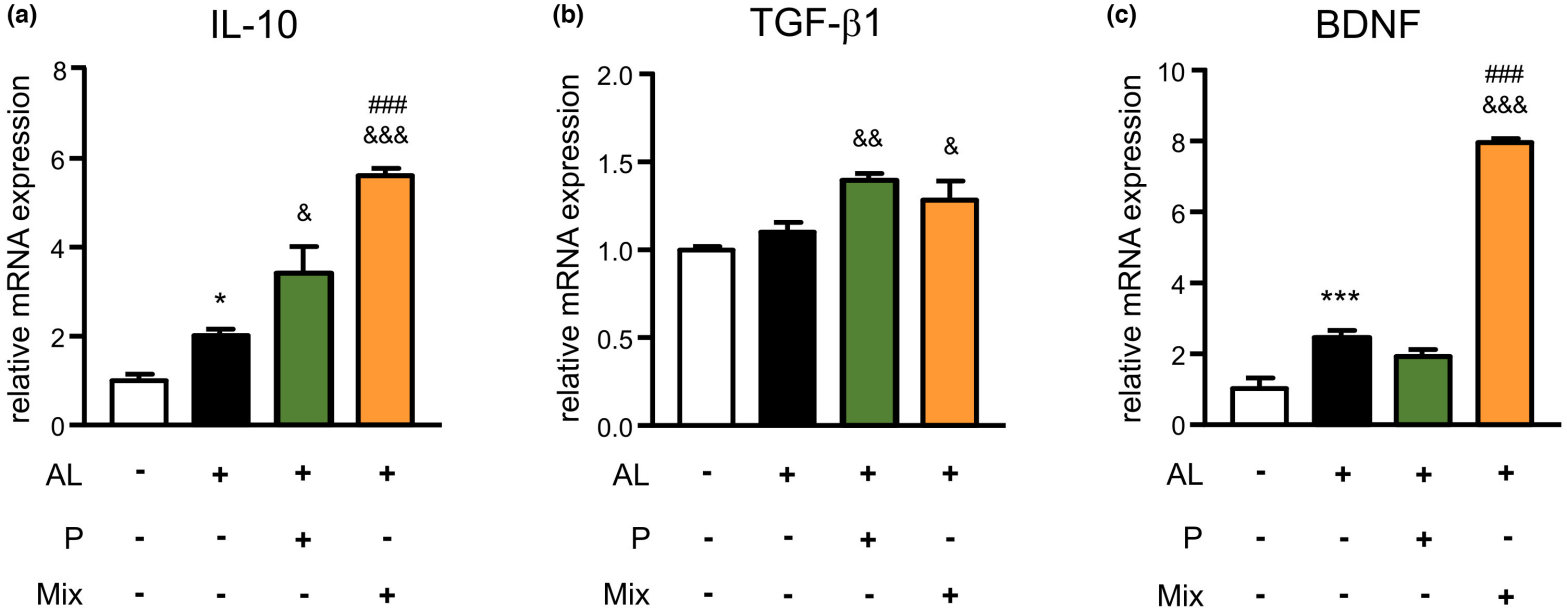
Mixture promoted the expression of anti‐inflammatory genes in microglia during exposure to Aβ and PgLPS. The mRNA expression of IL‐10 (a), TGFβ1 (b), and BDNF (c) at 1 h after exposure to Aβ and PgLPS (AL) with or without pretreatment with propolis (P) or mixture (Mix). Each column and bar represents the mean ± SD (*n* = 3, each). Asterisks indicate a statistically significant difference from the value in the control group (*p < .01, ***p < .001, one‐way ANOVA). Swords indicate a statistically significant difference from the value in the Aβ and PgLPS‐exposed group (^&^ *p* < .05, ^&&^ *p* < .01, ^&&&^ *p* < .001, one‐way ANOVA). Hash marks indicate a statistically significant difference from the value in the propolis and mixture group (^###^ *p* < .001, one‐way ANOVA).

Next, we investigated the effects of the mixture on anti‐inflammatory gene expression in AL‐exposed microglia. As shown in Figure 5, in comparison to control cells, the mRNA expression of IL‐10 and BDNF was raised in the AL‐exposed MG6 cells at 1 h after exposure to AL (2‐fold and 2.4‐fold rises). In comparison to the AL‐exposed MG6 cells, propolis alone significantly raised the mRNA expression of IL‐10 (3.4‐fold rise) and TGFβ1 (1.4‐fold rise), but not BDNF, in MG6 cells at 1 h after exposure to AL. The mixture significantly raised the mRNA expression of IL‐10, TGFβ1, and BDNF in MG6 cells at 1 h (5.6‐fold, 1.3‐fold, and 7.7‐fold rises) and lasted until 3 h after exposure to AL (data not shown). Notably, the mixture significantly increased the expression of IL‐10 and BDNF (but not TGFβ1) at 1 h compared with that of propolis alone in the AL‐exposed MG6 cells (1.6‐fold and 4.1‐fold rises). In comparison to control cells, the mRNA expression levels of IL‐10 and BDNF were increased at 1 h after exposure to AL (2‐fold and 2.4‐fold rises). In comparison to the AL‐exposed microglia, propolis alone significantly increased the mRNA expression of IL‐10 (3.4‐fold rise) and TGFβ1 (1.4‐fold rise), but not BDNF, in MG6 cells at 1 h after exposure to AL. The mixture significantly upregulated the mRNA expression of IL‐10, TGFβ1, and BDNF in MG6 cells at 1 h (5.6‐fold, 1.3‐fold, and 7.7‐fold increase) and lasted until 3 h after exposure to AL (data not shown). Notably, the mixture significantly promoted the expression of IL‐10 and BDNF (but not TGFβ1) at 1 h compared with that of propolis alone in the AL‐exposed MG6 cells (1.6‐fold and 4.1‐fold increases). In comparison to control cells, mRNA expression of IL‐10 was raised in BV2 cells exposed to AL (2‐fold rise) (Figure 4d,e). The mRNA expression of BDNF was also raised at 1 h after exposure to AL (1.46‐fold rise). Propolis alone significantly increased the mRNA expression of IL‐10 (2.26‐fold rise) and BDNF (2.77‐fold rise) in AL‐exposed BV2 cells. The mixture significantly increased the mRNA expression of IL‐10 and BDNF in AL‐exposed BV2 cells (5.8‐fold and 4.24‐fold rises). Furthermore, in comparison to propolis alone, the mixture significantly promoted the expression of IL‐10 and BDNF in the AL‐exposed BV2 cells at 1 h (2.56‐fold and 1.53‐fold rises) (Figure 4d,e). These observations demonstrate that the mixture promotes neuroprotective polarization of microglia during exposure to Aβ_1‐42_ and TLR ligands.

### 3.5 The mixture reduced NF‐κB activation in microglia during exposure to soluble Aβ and PgLPS

**FIGURE 6:**
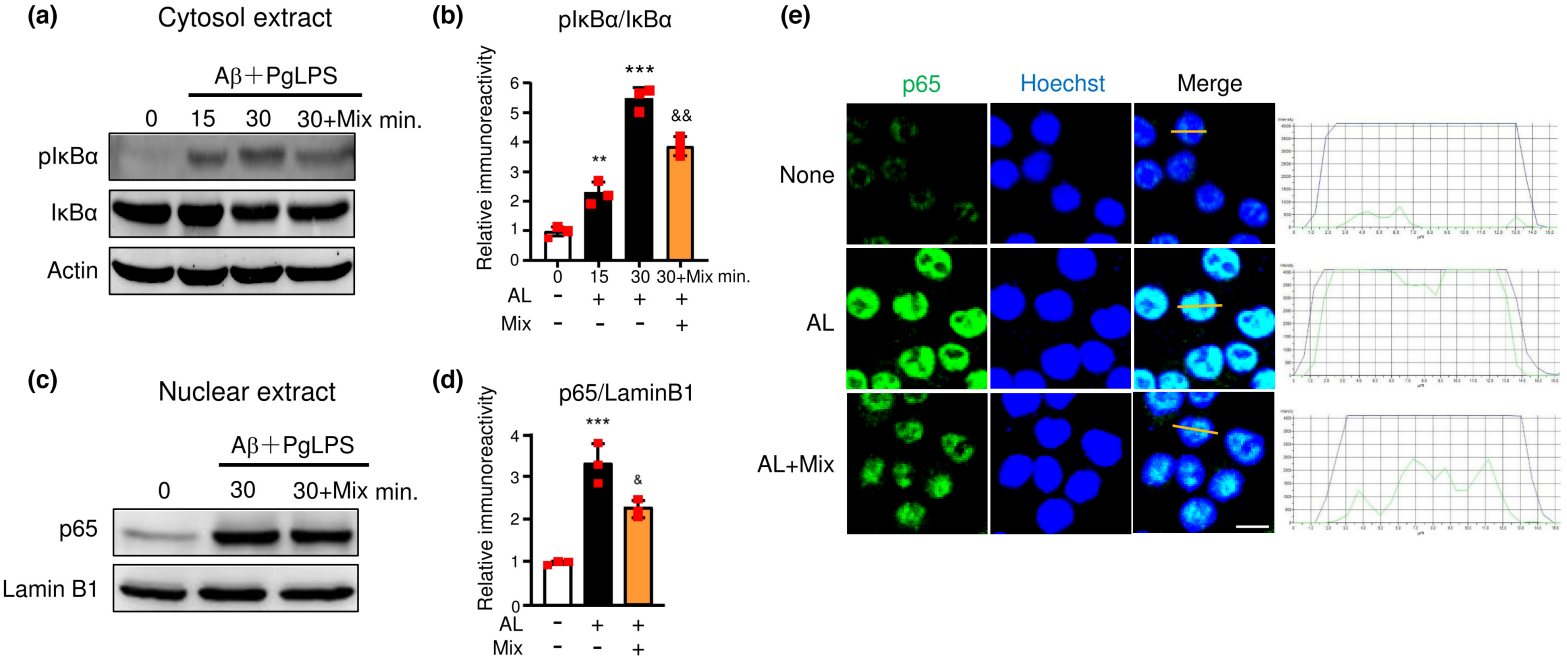
Mixture reduced NF‐κB activation in microglia during exposure to Aβ and PgLPS. (a) Time course of pIκBα and IκBα protein expression in cytosol extracts of microglia after exposure to Aβ and PgLPS (AL) with or without pretreatment with the mixture (Mix). (b) Quantitative analyses of immunoblots in (a). (c) p65 protein expression in the nuclear extract of microglia at 30 min after exposure to Aβ and PgLPS (AL) with or without pretreatment with the mixture (Mix). (d) Quantitative analysis of immunoblots in (c). (e) Immunofluorescent CLMS images indicating the nuclear translocation of p65 (green) in MG6 cells with Hoechst‐stained nuclei (blue) after exposure to AL for 1 h. Scale bar, 10 μm. Each column and bar represents the mean ± SD (*n* = 3, each). Asterisks indicate a statistically significant difference from the value at 0 min (*p < .01, ***p < .001, one‐way ANOVA). Swords indicate a statistically significant difference from the value in the Aβ and PgLPS‐exposed group (^&&^ *p* < .01, one‐way ANOVA).

We then investigated the outcomes of the mixture on NF‐κB activation in microglia after exposure to AL because NF‐κB critically controls the transcription of TNF‐α, IL‐1β, and IL‐6 (Liu et al., 2017). In comparison to control cells, IκBα phosphorylation in MG6 cells was upregulated from 15 min, with the increase reaching a statistically significant level at 30 min after exposure to AL (2.3‐fold rise at 15 min, 5.5‐fold rise at 30 min, Figure 6a,b). The mixture significantly reduced the elevated IκBα phosphorylation in AL‐exposed MG6 cells (29.2% inhibition at 30 min; Figure 6a,b). In comparison to control cells, p65 nuclear translocation was induced in MG6 cells at 30 min after AL exposure (3.3‐fold rise; Figure 6c,d). The mixture significantly reduced the p65 nuclear translocation in AL‐exposed MG6 cells (32.1% inhibition, *p* = .0109; Figure 6c,d). The original protein expression of NF‐κB activation for statistical analyses is shown in Figure S9. Immunofluorescent staining confirmed the increase in nuclear p65 localization in MG6 cells and BV2 cells at 1 h after AL exposure, and pretreatment with the mixture markedly reduced the AL‐increased nuclear p65 localization in both MG6 cells and BV2 cells (Figure 6e, Figure 4c). These results demonstrate that the mixture suppresses NF‐κB activation in microglia during exposure to Aβ_1‐42_ and TLR ligands.

### 3.6 The mixture induced Nrf2 activation in microglia during exposure to soluble Aβ and PgLPS

**FIGURE 7:**
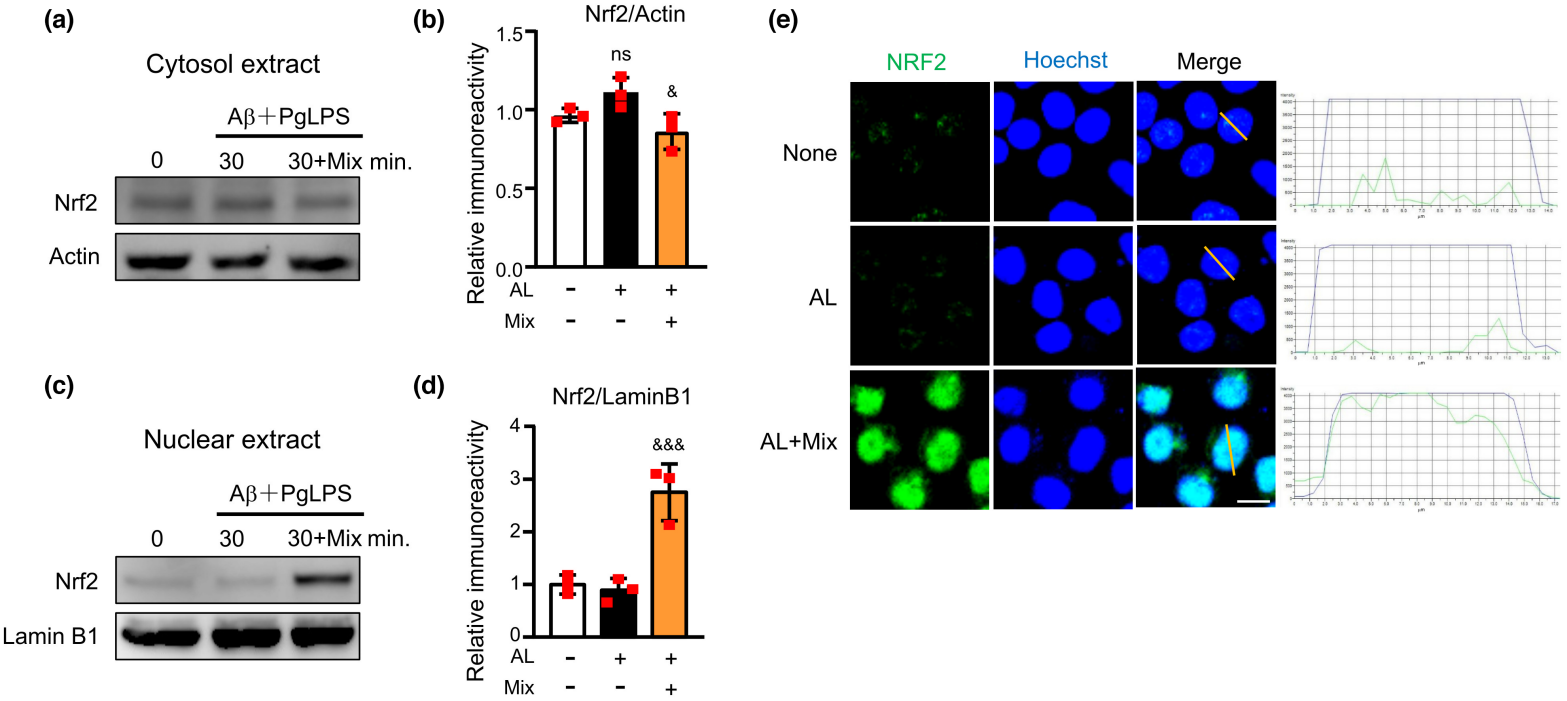
Mixture elevated Nrf2 activation in microglia during exposure to Aβ and PgLPS. (a) Nrf2 protein expression in the cytosol extract of microglia at 30 min after exposure to Aβ and PgLPS (AL) with or without pretreatment with the mixture (Mix). (b) Quantitative analyses of immunoblots in (A). Each column and bar represents the mean ± SD (*n* = 3, each). Swords indicate a statistically significant difference from the value in the Aβ and PgLPS‐exposed group (^&&^ *p* < .01, one‐way ANOVA). (C) Nrf2 protein expression in the nuclear extract of microglia at 30 min after exposure to Aβ and PgLPS (AL) with or without pretreatment with the mixture (Mix). (d) Quantitative analyses of immunoblots in (c). (e) Immunofluorescent CLMS images indicating the nuclear translocation of Nrf2 (green) in MG6 cells with Hoechst‐stained nuclei (blue) after exposure to AL for 1 h. Scale bar, 10 μm. Each column and bar represents the mean ± SD (*n* = 3, each). Swords indicate a statistically significant difference from the value in the Aβ‐ and PgLPS‐exposed group (^&&^ *p* < .01, one‐way ANOVA).

We then examined the effects of the mixture on Nrf2 activation in microglia after exposure to AL, because NrF2 is a transcription factor for attenuating inflammation (Cui et al., 2021; Okorji et al., 2016). Compared to control cells, the protein levels of Nrf2 in the cytosol were raised at 30 min after exposure to AL but did not reach significant levels (1.02‐fold rise at 30 min; Figure 7a,b); however, the Nrf2 nuclear translocation was not elevated in MG6 cells at 30 min after exposure to AL (Figure 7c,d). Compared to the AL‐exposed MG6 cells, the mixture significantly promoted Nrf2 nuclear translocation in the AL‐exposed microglia (2.08‐fold rise; Figure 7c,d). The original protein expression of Nrf2 activation for statistical analyses is shown in Figure S10. Immunofluorescent staining demonstrated that there was no increase in Nrf2 localization in MG6 cells or BV2 cells at 1 h after AL exposure. In contrast, nuclear Nrf2 localization was dramatically increased in AL‐exposed MG6 cells and BV2 cells that were pretreated with the mixture (Figure 7e, Figure 4f). These results demonstrate that the mixture induces Nrf2 activation during exposure to Aβ_1‐42_ and TLR ligands.

### 3.7 The mixture prevented mitochondrial dysfunction in microglia during exposure to soluble Aβ and PgLPS

**FIGURE 8:**
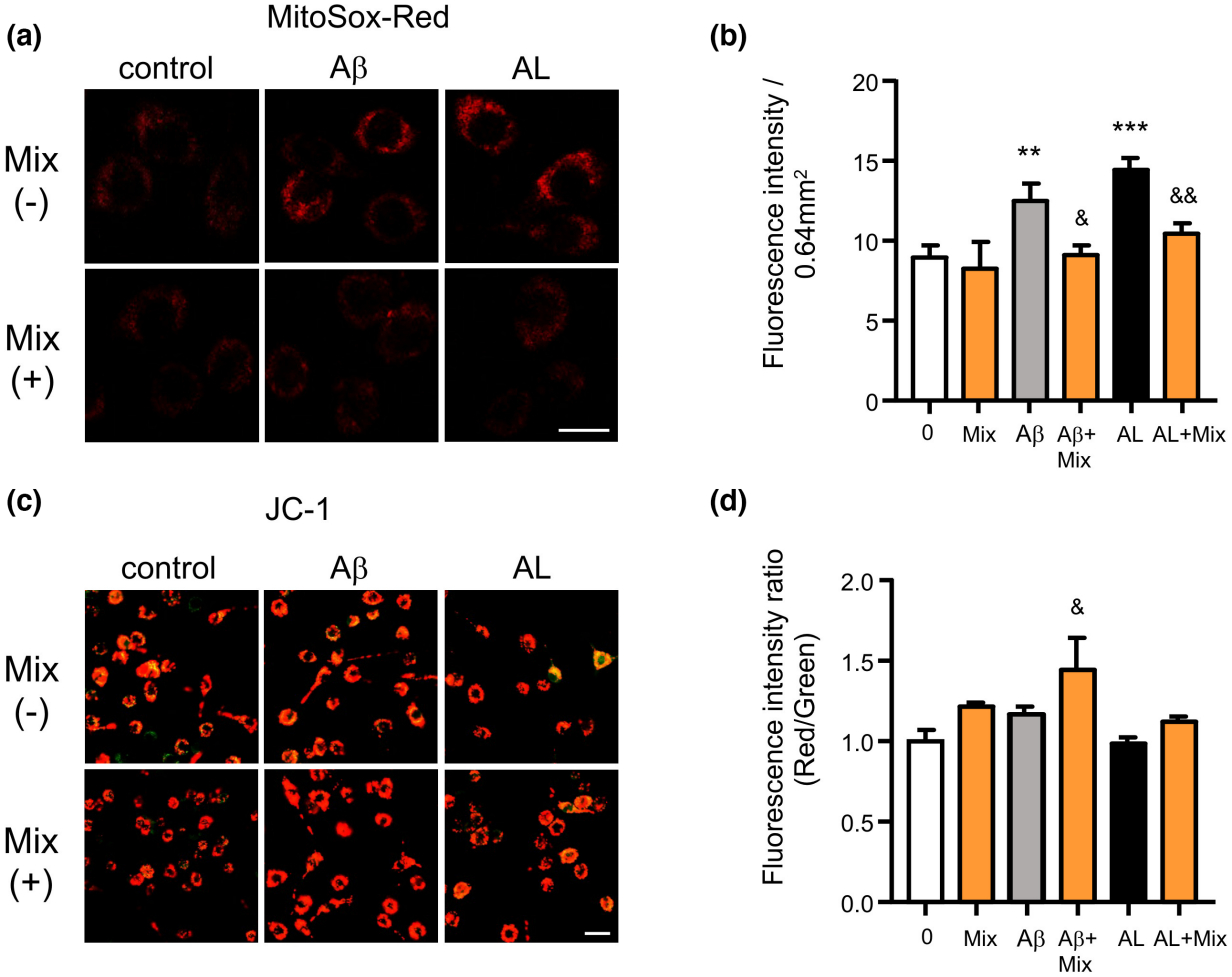
Mixture prevented mitochondrial dysfunction in microglia during exposure to Aβ and PgLPS. (a) Fluorescent images of MitoSOX Red fluorescence signals in MG6 cells at 1 h after exposure to either Aβ alone (a) or Aβ and PgLPS (AL) in the presence or absence of the mixture (Mix). Scale bar = 10 μm. (b) Quantitative analyses of the MitoSOX Red fluorescence signal intensity in (a). (c) Fluorescent images of JC‐1 fluorescence signals in MG6 cells at 1 h after exposure to either Aβ (a) or exposure to Aβ and PgLPS (AL) in the presence or absence of the mixture. Scale bar = 10 μm. (d) Quantitative analyses of the fluorescence intensity ratio (red/green) in (c). Each column and bar represents the mean ± SD (*n* = 4 each). An asterisk indicates a statistically significant difference from the value in the control group (**p < .01, ***p < .001, one‐way ANOVA). Swords indicate a statistically significant difference from the value in the Aβ or AL group (^&^ *p* < .05, ^&&^ *p* < .01, one‐way ANOVA).

We further examined the effects of the mixture on the mitochondrial functions of microglia after exposure to AL because microglial mitochondria are susceptible to oxidative damage, which regulates their functions (Nakanishi & Wu, 2009; Takashi et al., 2020). Mitochondrial ROS production in microglia was detected by the MitoSOX Red probe (Wu, Zhu, et al., 2013). In comparison to control cells, the mean immunofluorescence intensity (IFI) of MitoSOX Red oxidation was significantly raised in MG6 microglia at 1 h after exposure to Aβ_1‐42_ or AL (1.39‐fold and 1.61‐fold increase; Figure 8a,b), indicating that the generation of mitochondrial ROS in the microglia is elevated during exposure to Aβ_1‐42_ and AL. It is noted that the IFI of MitoSOX Red in AL‐exposed MG6 cells was higher than that in Aβ_1‐42_‐exposed MG6 cells (15.58% increased; Figure 8a,b). The mixture significantly reduced the mean IFI of MitoSOX Red in both Aβ_1‐42_ and AL‐exposed MG6 cells (27.03% and 36.87% decreased; Figure 8a,b). These results demonstrate that the mixture inhibited Aβ_1‐42_ and AL‐caused mitochondrial ROS generation in microglia. The mitochondrial membrane potentials (MMP) of microglia were detected using a JC‐1 kit. In comparison to control cells, the mean IFI ratio (Red/Green) was not significantly decreased in MG6 microglia at 1 h after exposure to Aβ_1‐42_ or AL (Figure 8c,d), indicating that the MMP of microglia is not damaged by exposure to low concentrations of Aβ or AL in this in vitro model. Pretreatment with the mixture increased the immunofluorescence intensity ratio in control and AL‐exposed MG6 cells (13.9% and 21.8% increased; Figure 8c,d), and significantly increased that of in Aβ_1‐42_‐exposed MG6 cells (23.7%; Figure 8c,d). The results demonstrate that the mixture improves the MMP of microglia.

## 4 DISCUSSION

**FIGURE 9:**
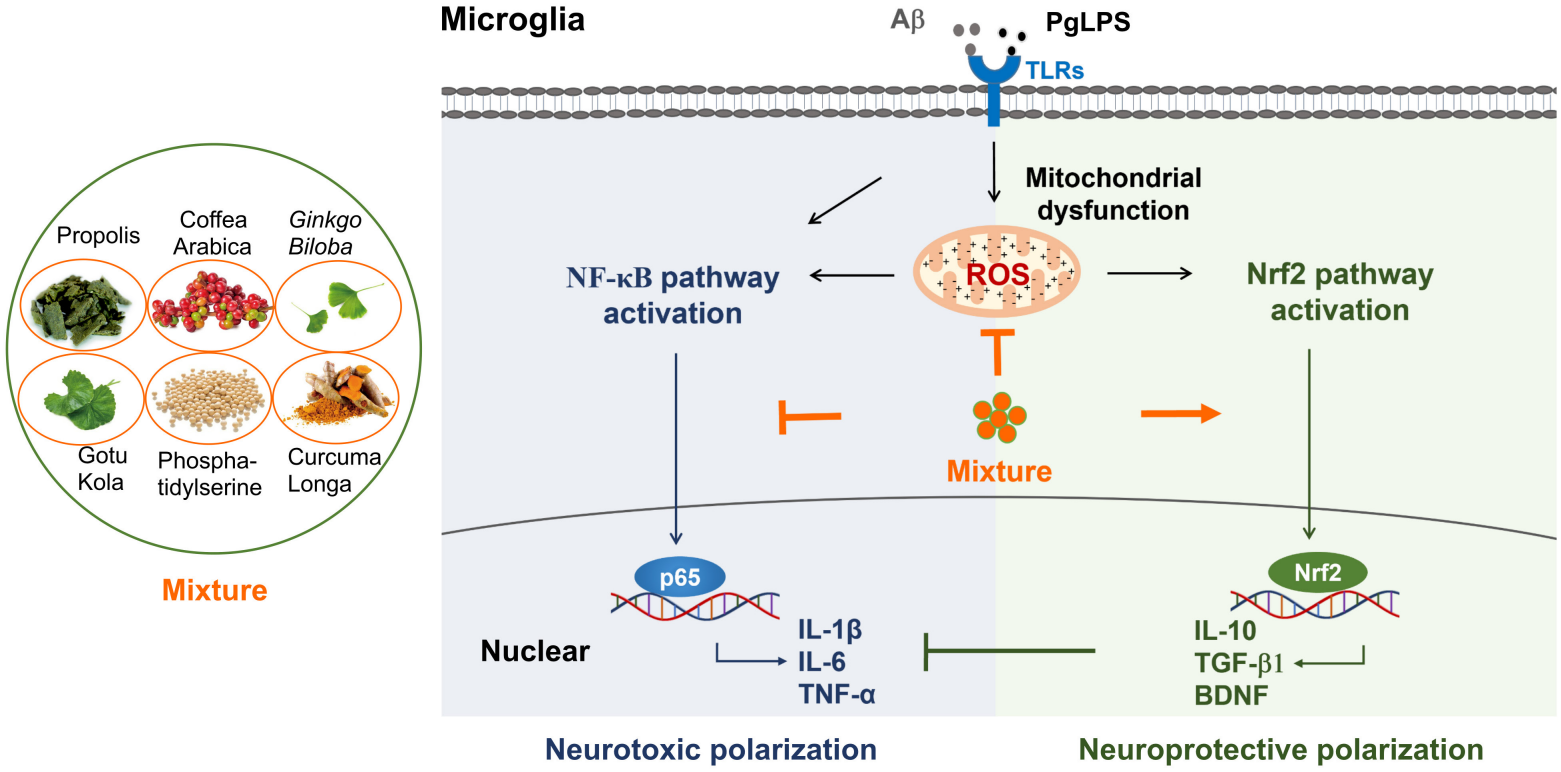
A schematic representation of the effects and novel molecular mechanisms of the mixture in facilitating the anti‐inflammatory potency of microglia during exposure to Aβ and PgLPS. Exposure to Aβ and PgLPS induces NF‐κB pathway activation to provoke the transcription of TNF‐α, IL‐1β, and IL‐6. Exposure to Aβ and PgLPS induces mitochondrial ROS generation to cause Nrf2 pathway activation. The mixture inhibits NF‐κB activation, resulting in the suppression of the expression of TNF‐α, IL‐1β, and IL‐6. This promotes Nrf2 nuclear translocation, resulting in the hastening of the expression of IL‐10, TGFβ1, and BDNF. This inhibits mitochondrial ROS generation and improves mitochondrial membrane potentials, resulting in the prevention of mitochondrial dysfunction in microglia during exposure to Aβ and PgLPS.

The original findings of this study are that the mixture of propolis, CA, GK, PS, GB, and CL synergistically reduces the neurotoxic polarization of microglia during exposure to Aβ_1‐42_ and TLR ligands. The molecular mechanisms underlying the beneficial effects of the mixture are dependent on modulating NF‐κB/Nrf2 pathways and improving mitochondrial functions (summarized in Figure 9). To our knowledge, this is the initial research to show the synergistic outcomes of natural ingredients on microglial polarization during exposure to Aβ_1‐42_ and TLR ligands.

Neuroinflammation facilitates the initiation and pathological processes of AD (Heneka et al., 2015). In vitro models are useful for exploring the direct effects of ingredients on functional targets of cells. We attempted to generate a microglia in vitro model that mimics microglia in the environment of the AD brain because microglia are critical contributors to neuroinflammation in AD, the most common neurodegenerative disorder with cognitive decline (Tran et al., 2022). The response of microglia to Aβ depends on its form and amount. In culture systems, inflammatory responses in microglia are induced by soluble Aβ_1‐42_ at concentrations over 5 μM (Heurtaux et al., 2010; Quiroga et al., 2022). In this study, soluble Aβ_1‐42_ at a concentration of 1 μM did not affect TNF‐α expression at either the transcriptional level or the protein level in MG6 microglia (Figure 2). This was supported by a previous report showing that 1 μM of soluble Aβ_1‐42_ did not trigger any immune transcriptional responses in microglia (Quiroga et al., 2022). These findings suggest that a low concentration of soluble Aβ_1‐42_ is insufficient for inducing microglia‐related neuroinflammation in the AD brain. To our surprise, exposure to soluble Aβ_1‐42_ (1 μM) with PgLPS (ligand of TLR2 and TLR4) promptly unregulated the proinflammatory mediators in microglia (1 h after coexposure), indicating that multiple TLR ligands provoke the neurotoxic polarization of microglia in the surroundings, even with a low concentration of soluble Aβ. Considering that Aβ accumulates 20 years before clinical symptoms occur (Randall et al., 2012) and that PgLPS is localized in the autopsy brains of AD patients (Poole et al., 2013), microglia exposure to soluble Aβ_1‐42_ and PgLPS can be used as an microglia in vitro model that mimics microglia‐related neuroinflammation in the AD brain.

Microglia‐related neuroinflammation has been identified as a target for early intervention to prevent neurodegeneration. Combination therapy will be useful for intervening in the cognitive decline during neurodegenerative diseases and aging, in which multiple targets should be modulated. To develop a safe combination, it is necessary to determine the appropriate usage of ingredients based on the careful screening of each ingredient (Figures S1–S6). We found a mixture that could be safely used by ensuring that none of the components in the mixture had any cytotoxic effects on microglia (Figure 1). Propolis alone suppressed the upregulation of TNF‐α, IL‐1β, and IL‐6 in microglia by exposure to Aβ_1‐42_ and PgLPS, supporting the suppression effects of propolis on proinflammatory responses in hypoxia or PgLPS‐exposed microglia (Liu et al., 2013; Wu, Sun, et al., 2013; Wu, Zhu, et al., 2013). In comparison to propolis alone, the mixture had greater suppression effects on the AL‐upregulated TNF‐α and IL‐1β, demonstrating that the mixture synergistically reduced the neurotoxic polarization of microglia in the environment of the AD brain. The synergistic effects of the mixture on neurotoxic responses in microglia may be dependent on the comprehensive effects of individual ingredients because the production of IL‐1β and TNF‐α was inhibited by CA, GK, PS, GB, and CL (De Simone et al., 2003; Delerue et al., 2021; Huynh et al., 2002; Shi et al., 2015; Sowndhararajan et al., 2018; Wu, Sun, et al., 2013; Wu, Zhu, et al., 2013; Zhang et al., 2022). The reduction of TNF‐α and IL‐1β in microglia is extremely important for delaying the pathological process of AD because microglia‐produced IL‐1β promotes Aβ aggregation and microglia‐produced TNF‐α promotes tau phosphorylation in neurons (Jiang et al., 2021; Wu et al., 2017). The expression of IL‐10 and BDNF in microglia was upregulated at 1 h after exposure to Aβ_1‐42_ and PgLPS, indicating that microglia have neuroprotective ability during exposure to ligands of TLR with soluble Aβ_1‐42_. Propolis alone increased the expression of IL‐10 and TGFβ1 in Aβ_1‐42_ and PgLPS‐exposed microglia, supporting the anti‐inflammatory effects of propolis (Zhu et al., 2018). The synergistic effects of the mixture on IL‐10 upregulation (5.6‐fold increase in MG6 cells and 5.8‐fold increase in BV2 cells) are considered to represent the additive effects of GK (Masola et al., 2018) and CL (Porro et al., 2019). Interestingly, the mixture dramatically increased the expression of BDNF in microglia during exposure to Aβ_1‐42_ and PgLPS (7.7‐fold increase in MG6 cells and 4.24‐fold increase in BV2 cells). The BDNF‐promoting effect of the mixture is considerably dependent on the synergistic effects of GK (Sbrini et al., 2020), GB (Sadowska‐Krępa et al., 2017), PS, and CL (McDaniel et al., 2003; Okuda et al., 2019). The elevation of BDNF in microglia by treatment with the mixture contributes to the prevention of cognitive decline because BDNF is majorly involved in neuroplasticity and the formation of learning and memory (Parkhurst et al., 2013). The greater modulatory effects of the mixture on microglia than those of propolis alone demonstrate that the mixture synergistically functions on microglial polarization. Taken together, the mixture shifts the neurotoxic microglia into neuroprotective ones during exposure to Aβ and PgLPS.

Multiple molecular mechanisms are considered to underlie the regulation of microglial function by the mixture. First, the mixture directly suppressed NF‐κB activation. This was confirmed by the finding that both IκBα phosphorylation and p65 nuclear translocation were significantly inhibited in AL‐exposed microglia (Figures 6 and 4c). The restraint of NF‐κB activity by the mixture contributes to changes in the microglia functional phenotype because NF‐κB signaling is crucial for regulating the phenotype of microglia (Kopitar‐Jerala, 2015). The restrained effects of the mixture on NF‐κB activation may be dependent on the synergistic effects of propolis, GK, GB, and CL, which inhibit NF‐κB activation (Ran et al., 2021; Seo et al., 2021; Wu, Sun, et al., 2013; Wu, Zhu, et al., 2013). Second, the mixture hastened Nrf2 activation directly. This possibility was confirmed by finding that the ingredient mixture remarkably promoted Nrf2 translocation into the nucleus of AL‐exposed microglia (Figures 7 and 4f). The contributions of Nrf2 to anti‐inflammation have been recognized because Nrf2‐deficient mice showed increased levels of IL‐6 and TNF‐α in response to neurotoxin exposure (Rojo et al., 2010), and Nrf2 binds in the proximity of IL‐1β and IL‐6 genes to reduce their transcription in macrophages (Kobayashi et al., 2016). The additive effects of CA and GK contribute to enhancing the effects of the ingredient mixture on Nrf2 activation (Liu et al., 2020; Matthews et al., 2019). Third, the mixture interferes with the crosstalk between NF‐κB and Nrf2 (Huang et al., 2022; Wardyn et al., 2015). The simultaneous suppression of NF‐κB activity and the promotion of Nrf2 activity by the mixture may contribute to shifting the microglia to neuroprotective phenotypes in AL‐exposed microglia because the absence of Nrf2 exaggerates NF‐κB activity by enhancing IκBα degradation (Rojo et al., 2010), while NF‐κB subunit p65 downregulates Nrf2 activity (Wardyn et al., 2015). Fourth, the mixture improves mitochondrial functions. The elevation of mitochondrial ROS in AL‐exposed microglia indicates that oxidative stress is induced by the activation of TLRs during exposure to low amounts of soluble Aβ_1‐42_. Low amounts of soluble Aβ_1‐42_ are not sufficient to directly induce microglia‐related neuroinflammation (Figure 2), but Aβ elevates oxidative stress in microglia, resulting in the sensitive response of PgLPS in promoting inflammatory responses (Figure 8). The reduction in oxidative stress by the mixture may help shift microglia into neuroprotective phenotypes. The hastening of Nrf2 nuclear translocation by the ingredient mixture may contribute to reducing oxidative stress in microglia because Nrf2 acts as a key regulator of equilibrium (Kerins & Ooi, 2018). As MMP is important for the generation of ATP (Wilkins et al., 2017), the improvement of MMP by the ingredient mixture may contribute to maintaining the quality of the mitochondria for energy production and the survival of microglia. Considering the involvement of TLR activation in mitochondrial ROS generation (Geng et al., 2015) and the core role of mitochondria in microglia‐related neuroinflammation (Agrawal & Jha, 2020), the prevention of mitochondrial dysfunction by the ingredient mixture may potentially contribute to the mitigation of neuroinflammation (Cenini & Voos, 2019). These observations indicate that the mixture of natural ingredients synergistically dampened microglia‐mediated neuroinflammation by modulating multiple targets. Dampening neuroinflammation by ingredient mixture will contribute to cognitive improvement in humans, as a recent clinical study reported that intake of a composite dietary supplement containing propolis, GB, PS, and curcumin for 12 weeks improved the cognitive function of mid‐ to senior‐age Japanese adults (Takashi et al., 2020). The concept of synergistic effects is supported by research that showed that the combined effect of nutrients suppresses the decline in muscle mass and physical function in humans (Yokoyama et al., 2017). Further experiments are needed to explore whether or not the ingredient mixture directly affects other brain cells, including astrocytes and neurons.

## 5 CONCLUSION

The present study demonstrated synergistic anti‐inflammatory and antioxidative effects of propolis, CA, GK, PS, GB, and CL on microglia during exposure to Aβ_1‐42_ and TLR ligands by modulating NF‐κB/Nrf2 activation and improving mitochondrial functions. Our study provides new evidence to support the idea that a combination of natural ingredients will be a useful measure for the prevention of cognitive decline in AD and aging.

## AUTHOR CONTRIBUTIONS

**Shuge Gui:** Conceptualization (supporting); data curation (equal); formal analysis (equal); methodology (supporting); writing – original draft (lead). **Junjun Ni:** Data curation (equal); methodology (equal); writing – review and editing (equal). **Shinsuke Mizutani:** Formal analysis (equal); methodology (supporting). **Norihiro Shigematsu:** Formal analysis (equal). **Hiroshi Nakanishi:** Formal analysis (equal). **Haruhiko Kashiwazaki:** Formal analysis (equal). **Zhou Wu:** Conceptualization (lead); methodology (equal); supervision (lead); writing – review and editing (lead).

## CONFLICT OF INTEREST STATEMENT

The authors declare that the research was conducted in the absence of any commercial or financial relationships that could be constructed as a potential conflict of interest.

## ETHICS STATEMENT

This study does not involve any human or animal testing.

## Supporting information


Appendix S1.

## Data Availability

The data that support the findings of this study are available from the corresponding author upon request.
