# Supplementary material for: A mixture of extracts from natural ingredients reduces the neurotoxic polarization of microglia via modulating NF‐κB/NF‐E2‐related factor 2 activation
Source: Food Sci Nutr. 2024 Feb 20;12(5):3745–58. doi: 10.1002/fsn3.4045 (PMC11077187; doi:10.1002/fsn3.4045)
Supplement: Supplementary file 1 — Appendix S1. [file FSN3-12-3745-s001.zip › Captions.docx]

Supplementary TABLE 1 Sequences of the primer pairs for RT–qPCR.

Supplementary FIGURE 1 Determination of the effect of propolis on microglial viability. (A) 24 h, (B) 48 h, and (C) 72 h after treatment with propolis. Asterisks indicate a statistically significant difference from the value in the control (0) group (*P < 0.05, **P < 0.01, ***P < 0.001, one-way ANOVA).

Supplementary FIGURE 2 Determination of Coffea arabica on microglial viability. (A) 24 h, (B) 48 h, and (C) 72 h after treatment with Coffea arabica. Asterisks indicate a statistically significant difference from the value in the control (0) group (*P < 0.05, **P < 0.01, ***P < 0.001, one-way ANOVA).

Supplementary FIGURE 3 Determination of the effect of Gotu kola on microglial viability. (A) 24 h, (B) 48 h, and (C) 72 h after treatment with Gotu kola. Asterisks indicate a statistically significant difference from the value in the control (0) group (*P < 0.05, **P < 0.01, ***P < 0.001, one-way ANOVA).

Supplementary FIGURE 4 Determination of phosphatidylserine on microglial viability. (A) 24 h, (B) 48 h, and (C) 72 h after treatment with phosphatidylserine. Asterisks indicate a statistically significant difference from the value in the control (0) group (*P < 0.05, **P < 0.01, ***P < 0.001, one-way ANOVA).

Supplementary FIGURE 5 Determination of Ginkgo biloba on microglial viability. (A) 24 h, (B) 48 h, and (C) 72 h after treatment with Ginkgo biloba. Asterisks indicate a statistically significant difference from the value in the control (0) group (*P < 0.05, **P < 0.01, ***P < 0.001, one-way ANOVA).

Supplementary FIGURE 6 Determination of Curcuma longa on microglial viability. (A) 24 h, (B) 48 h, and (C) 72 h after treatment with Curcuma longa. Asterisks indicate a statistically significant difference from the value in the control (0) group (*P < 0.05, **P < 0.01, ***P < 0.001, one-way ANOVA).

Supplementary FIGURE 7 Determination of Aβ or Aβ with PgLPS on microglial viability. (A) 24 h, (B) 48 h and (C) 72 h after treatment with Aβ or Aβ with PgLPS. There was no significant difference from the value in the control (0) group (one-way ANOVA).

Supplementary FIGURE 8 Determination of Aβ with PgLPS and Mix 2 on microglial viability. (A) 24 h, (B) 48 h, and (C) 72 h after treatment with Aβ or Aβ with PgLPS. There was no significant difference from the value in the control (0) group (one-way ANOVA).

Supplementary FIGURE 9 The protein expression of NF-κB activation for the statistical analyses. (A) Protein expression of pIκBα and IκBα pin cytosol extract of microglia after exposure to Aβ and PgLPS (AL) with or without pretreatment with mixture (Mix) for the statistical analyses in Figure 5b. (B) p65 protein expression in the nuclear extract of microglia at 30 min after exposure to Aβ and PgLPS (AL) with or without pretreatment with the mixture (Mix) for the statistical analyses in Figure 5d.

Supplementary FIGURE 10 Nrf2 protein expression for the statistical analyses. (A) Nrf2 protein expression in the cytosol extract of microglia at 30 min after exposure to Aβ and PgLPS (AL) with or without pretreatment with the mixture (Mix) for the statistical analyses in Figure 6b. (B) Nrf2 protein expression in the nuclear extract of microglia at 30 min after exposure to Aβ and PgLPS (AL) with or without pretreatment with the mixture (Mix) for the statistical analyses in Figure 6d.
