# Supplementary figures and images for: A mixture of extracts from natural ingredients reduces the neurotoxic polarization of microglia via modulating NF‐κB/NF‐E2‐related factor 2 activation
Source: Food Sci Nutr. 2024 Feb 20;12(5):3745–58. doi: 10.1002/fsn3.4045 (PMC11077187; doi:10.1002/fsn3.4045)

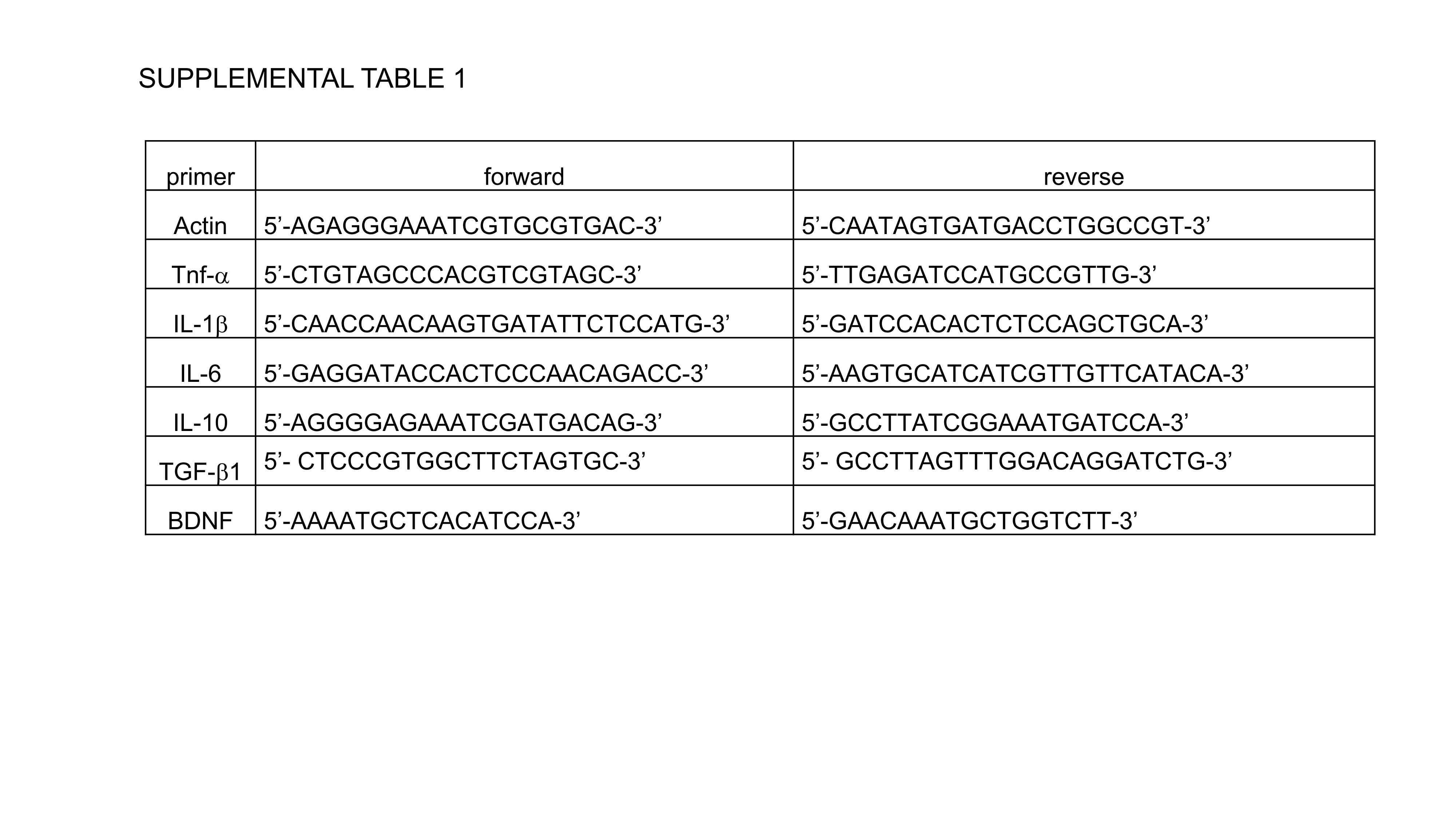

Supplement: Supplementary file 1 — Appendix S1. [file FSN3-12-3745-s001.zip › fsn34045-sup-0001-Table S1.tif]

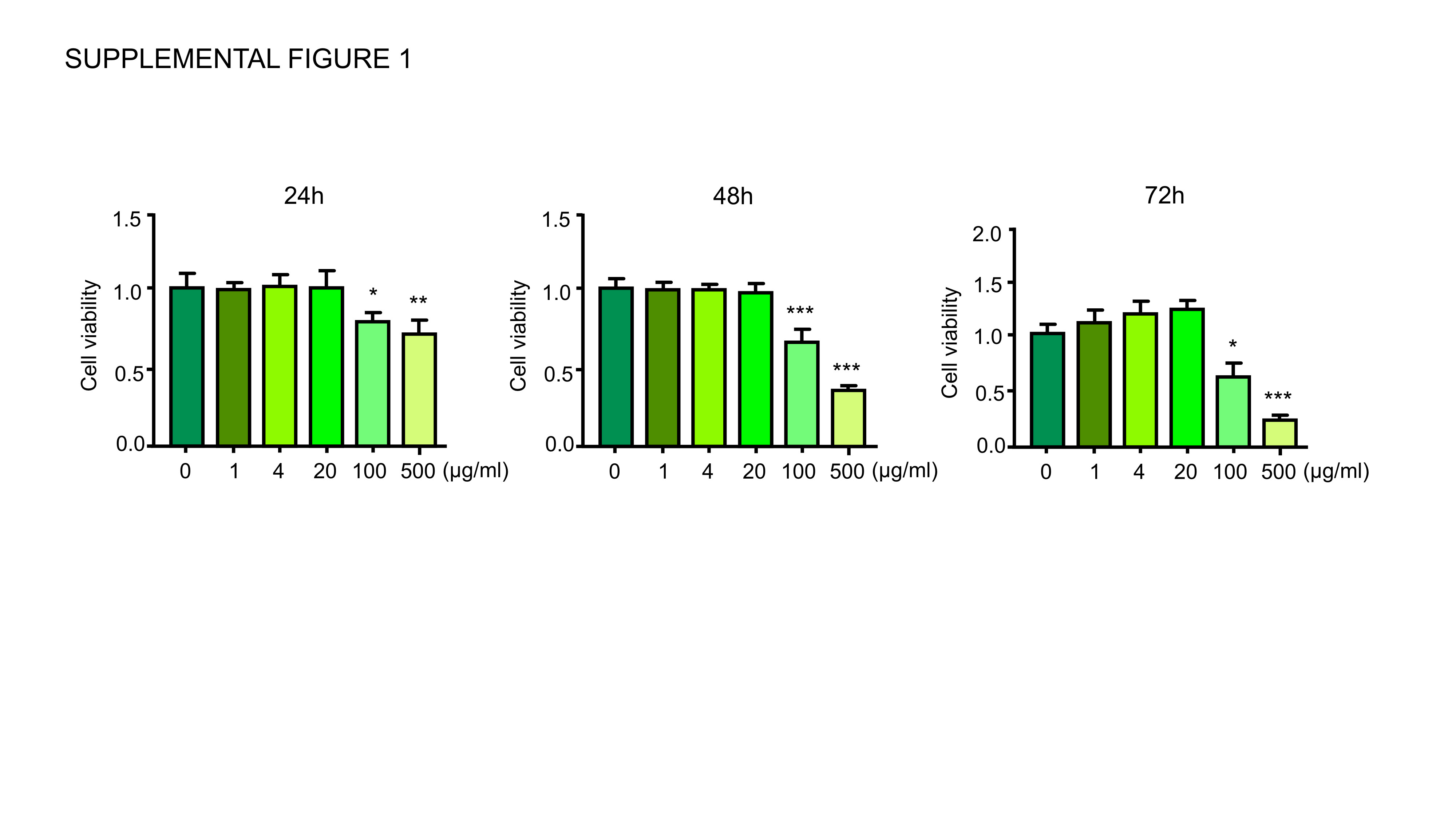

Supplement: Supplementary file 1 — Appendix S1. [file FSN3-12-3745-s001.zip › fsn34045-sup-0002-FigureS1.tif]

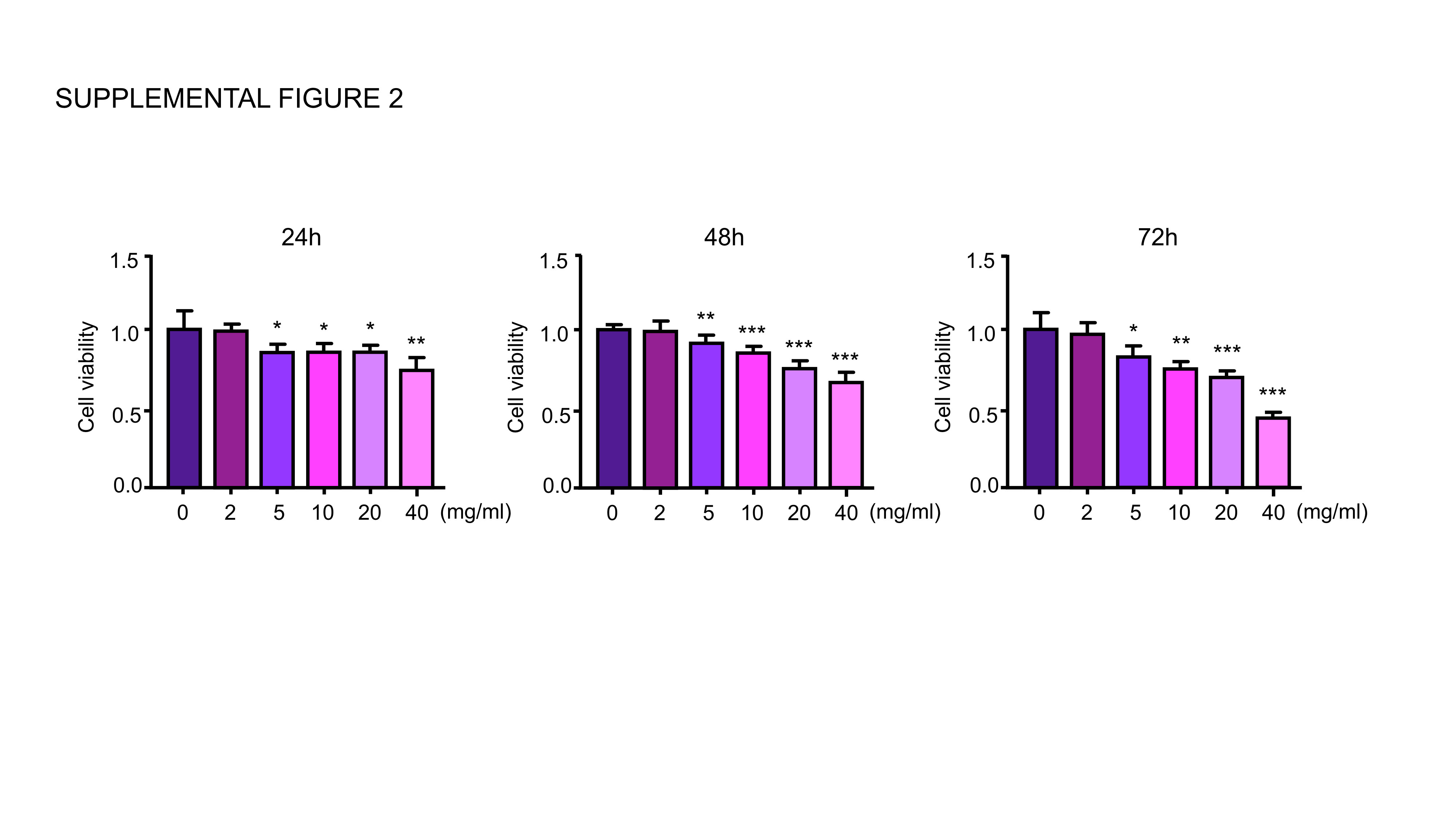

Supplement: Supplementary file 1 — Appendix S1. [file FSN3-12-3745-s001.zip › fsn34045-sup-0003-FigureS2.tif]

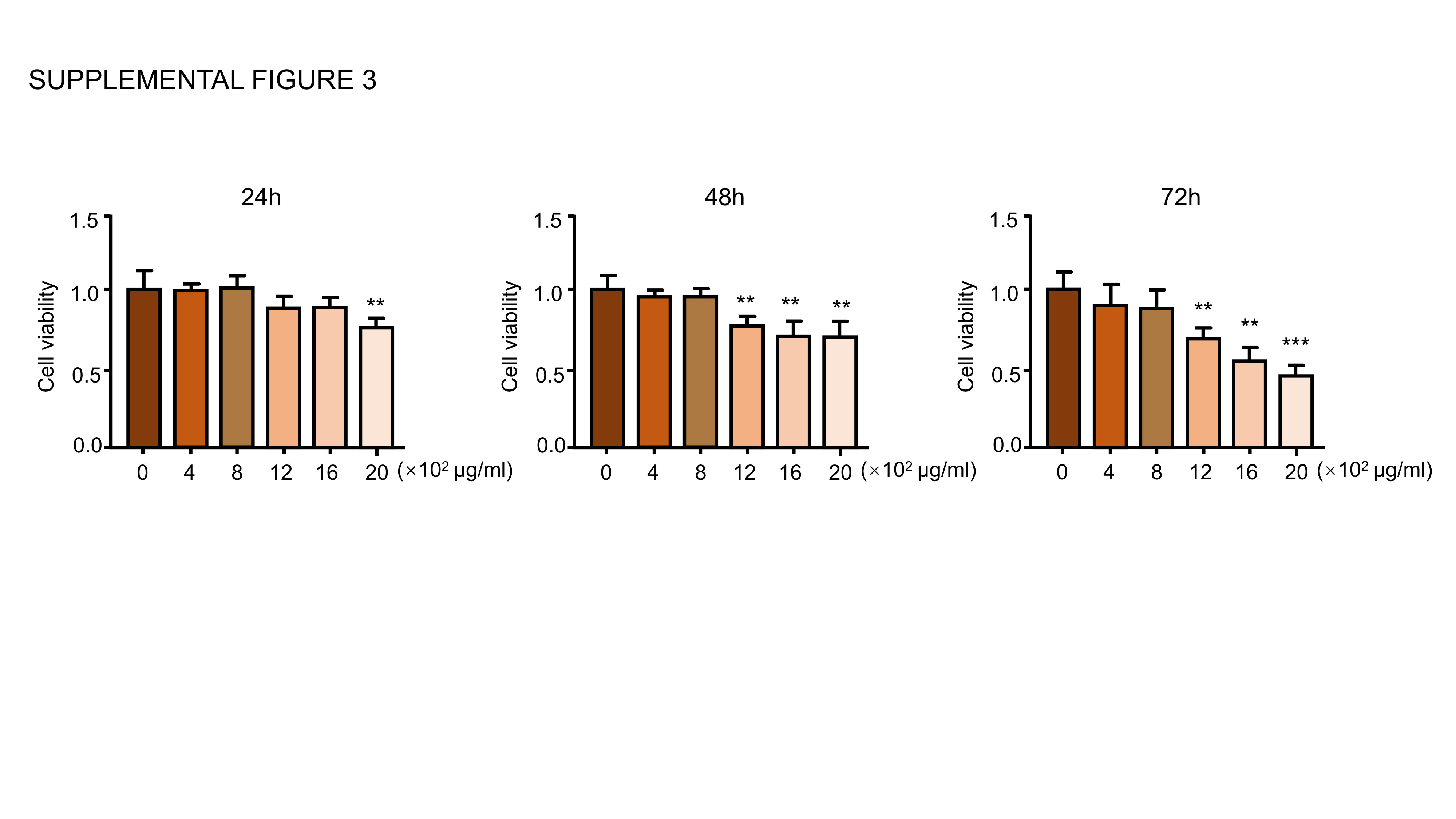

Supplement: Supplementary file 1 — Appendix S1. [file FSN3-12-3745-s001.zip › fsn34045-sup-0004-FigureS3.tif]

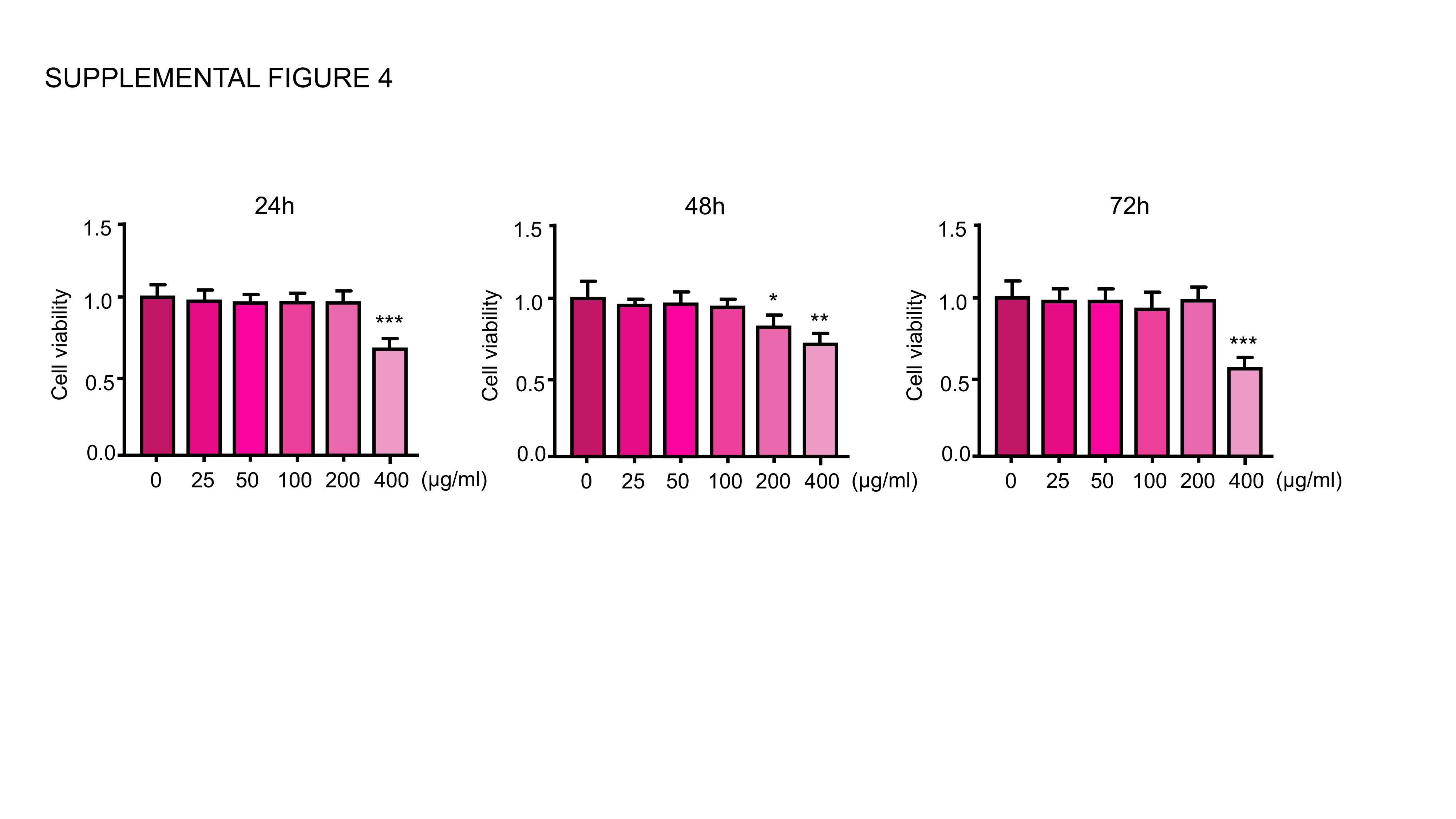

Supplement: Supplementary file 1 — Appendix S1. [file FSN3-12-3745-s001.zip › fsn34045-sup-0005-FigureS4.tif]

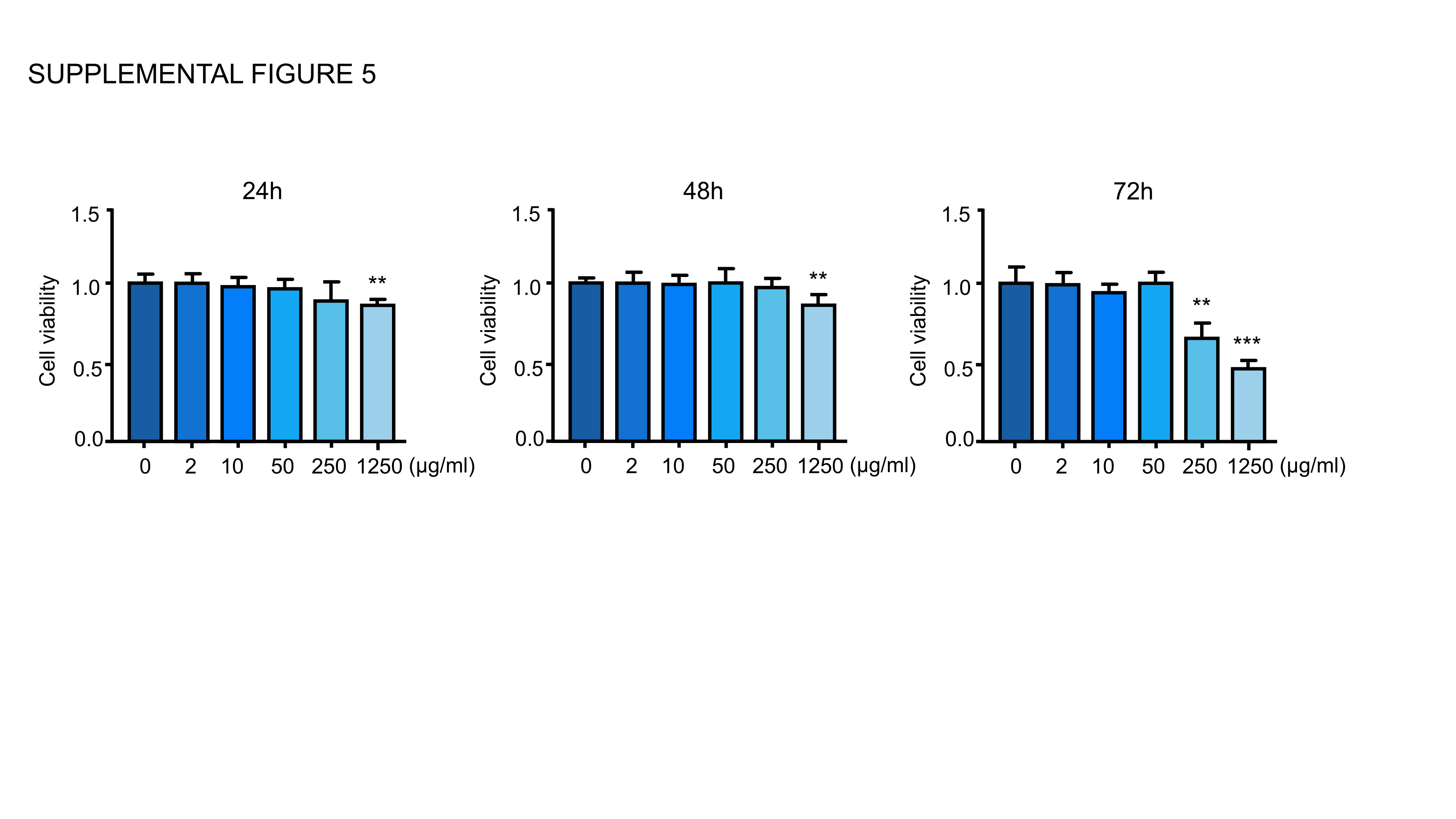

Supplement: Supplementary file 1 — Appendix S1. [file FSN3-12-3745-s001.zip › fsn34045-sup-0006-FigureS5.tif]

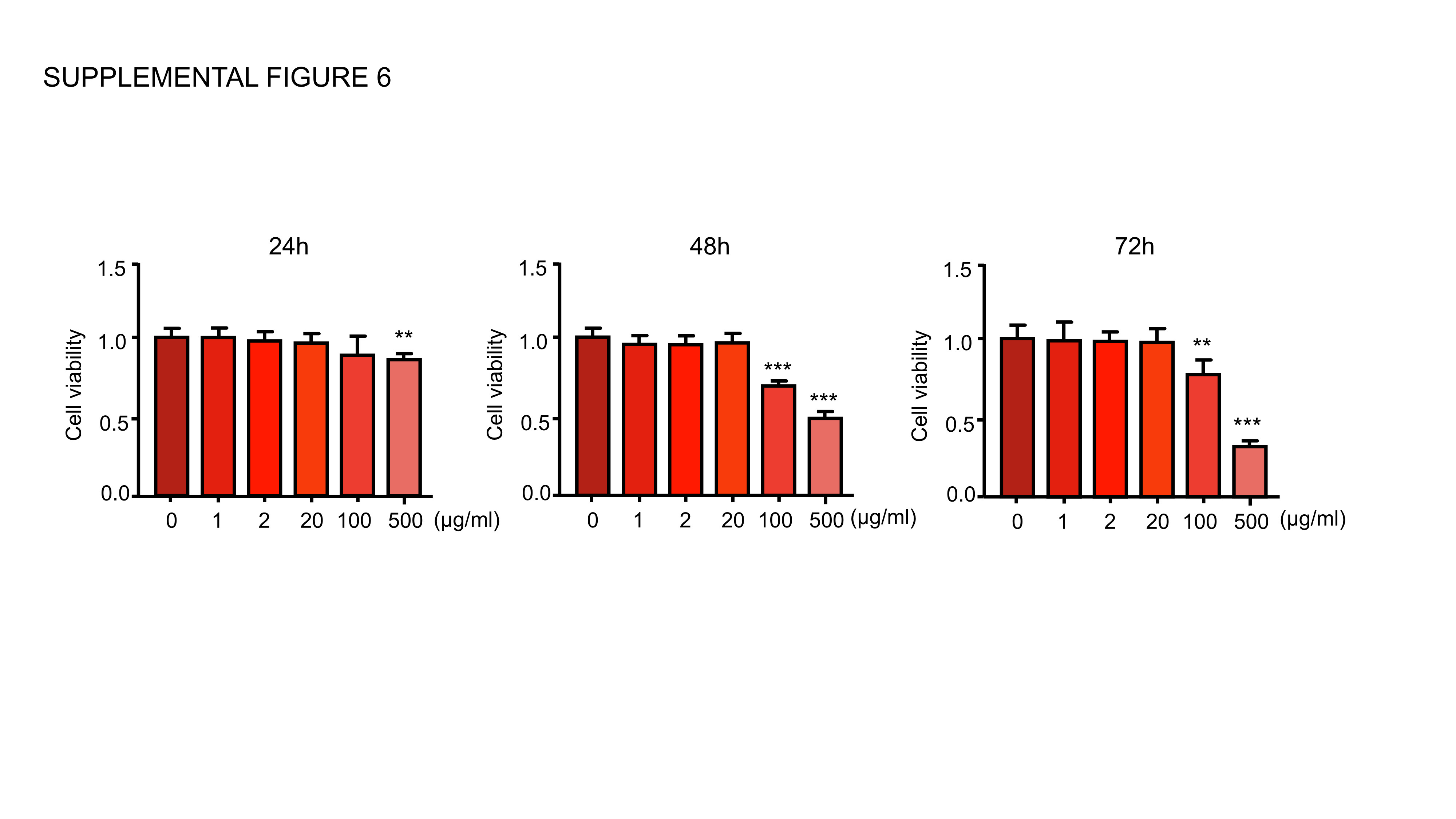

Supplement: Supplementary file 1 — Appendix S1. [file FSN3-12-3745-s001.zip › fsn34045-sup-0007-FigureS6.tif]

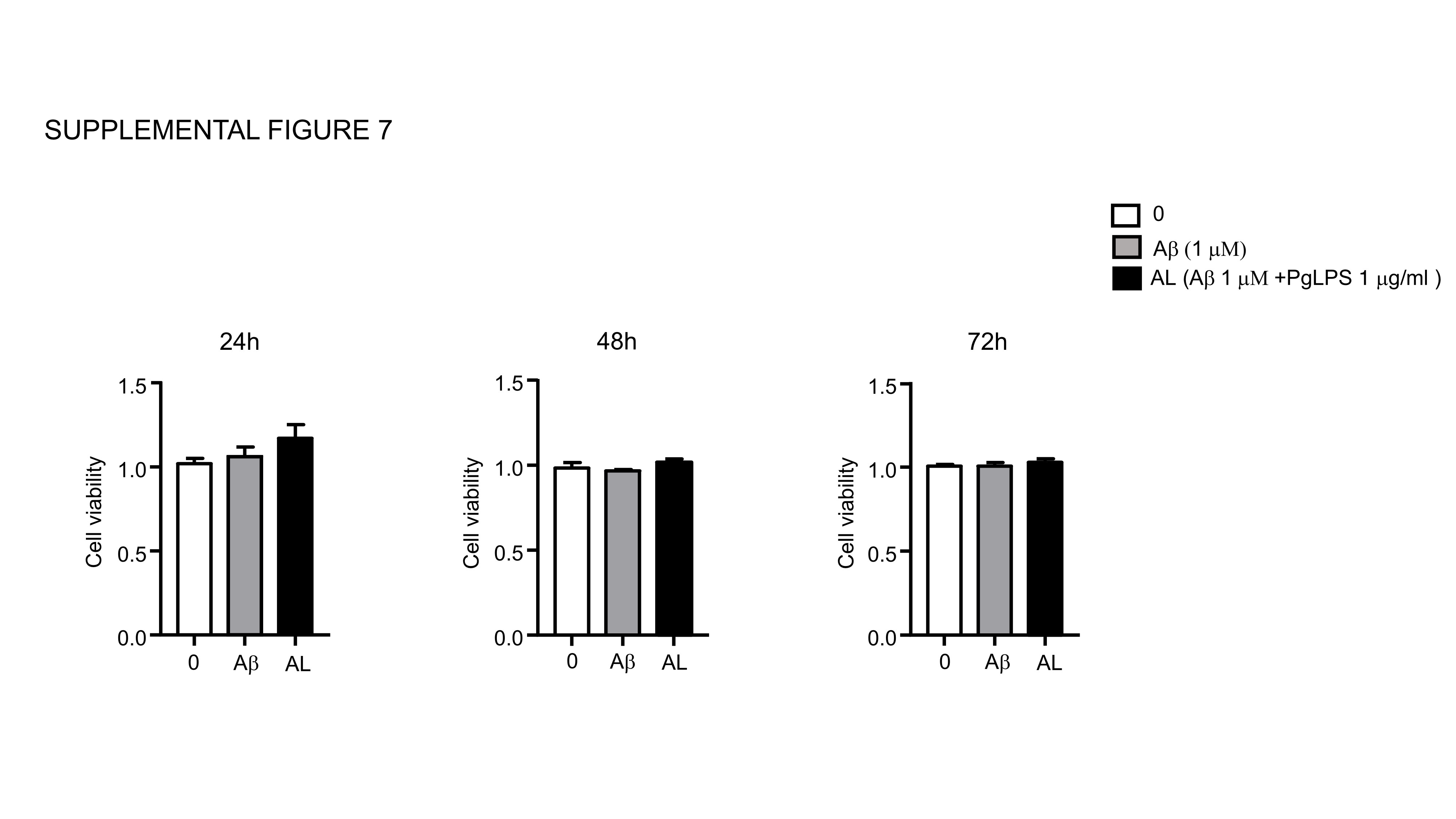

Supplement: Supplementary file 1 — Appendix S1. [file FSN3-12-3745-s001.zip › fsn34045-sup-0008-FigureS7.tif]

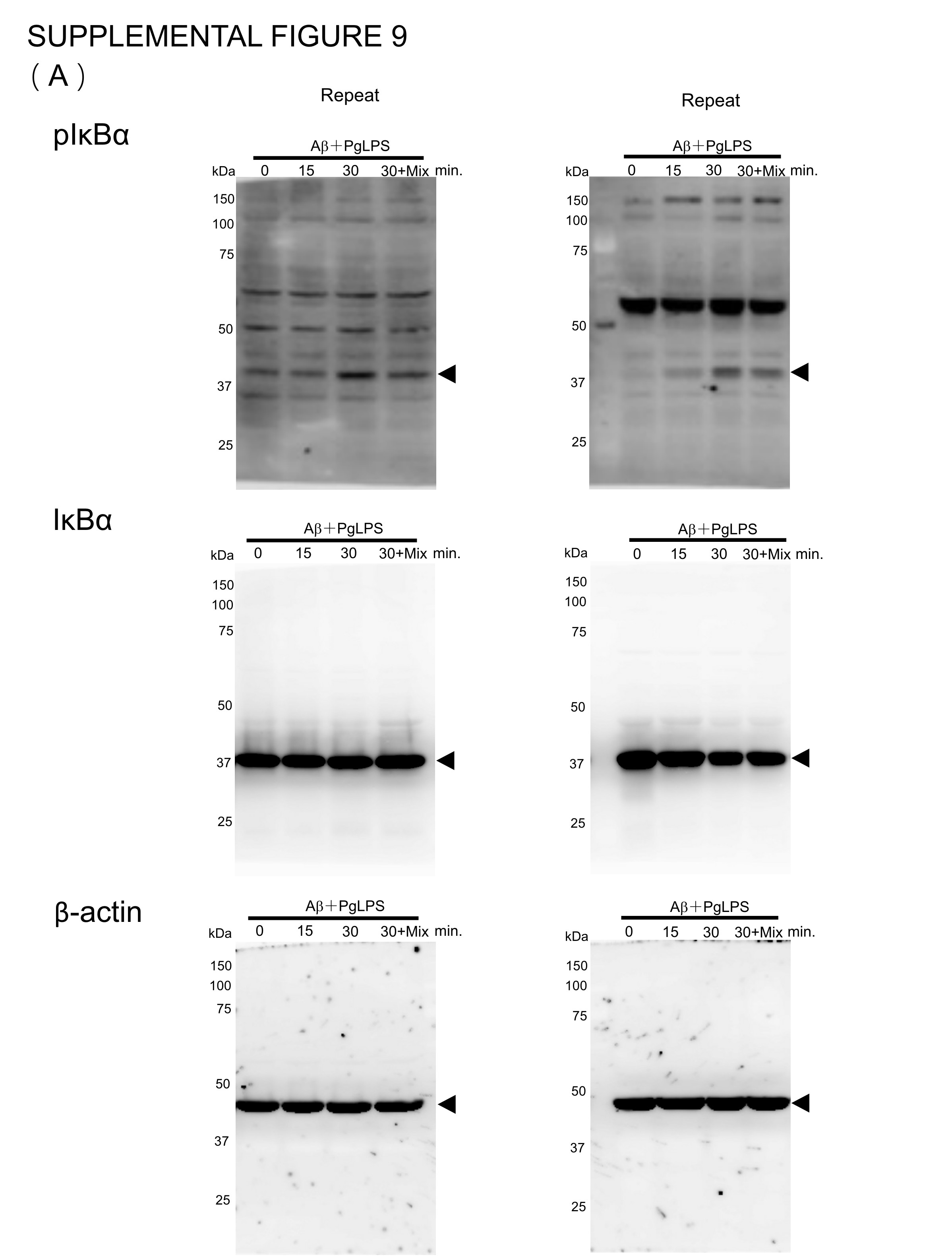

Supplement: Supplementary file 1 — Appendix S1. [file FSN3-12-3745-s001.zip › fsn34045-sup-0009-Figure 9A.tif]

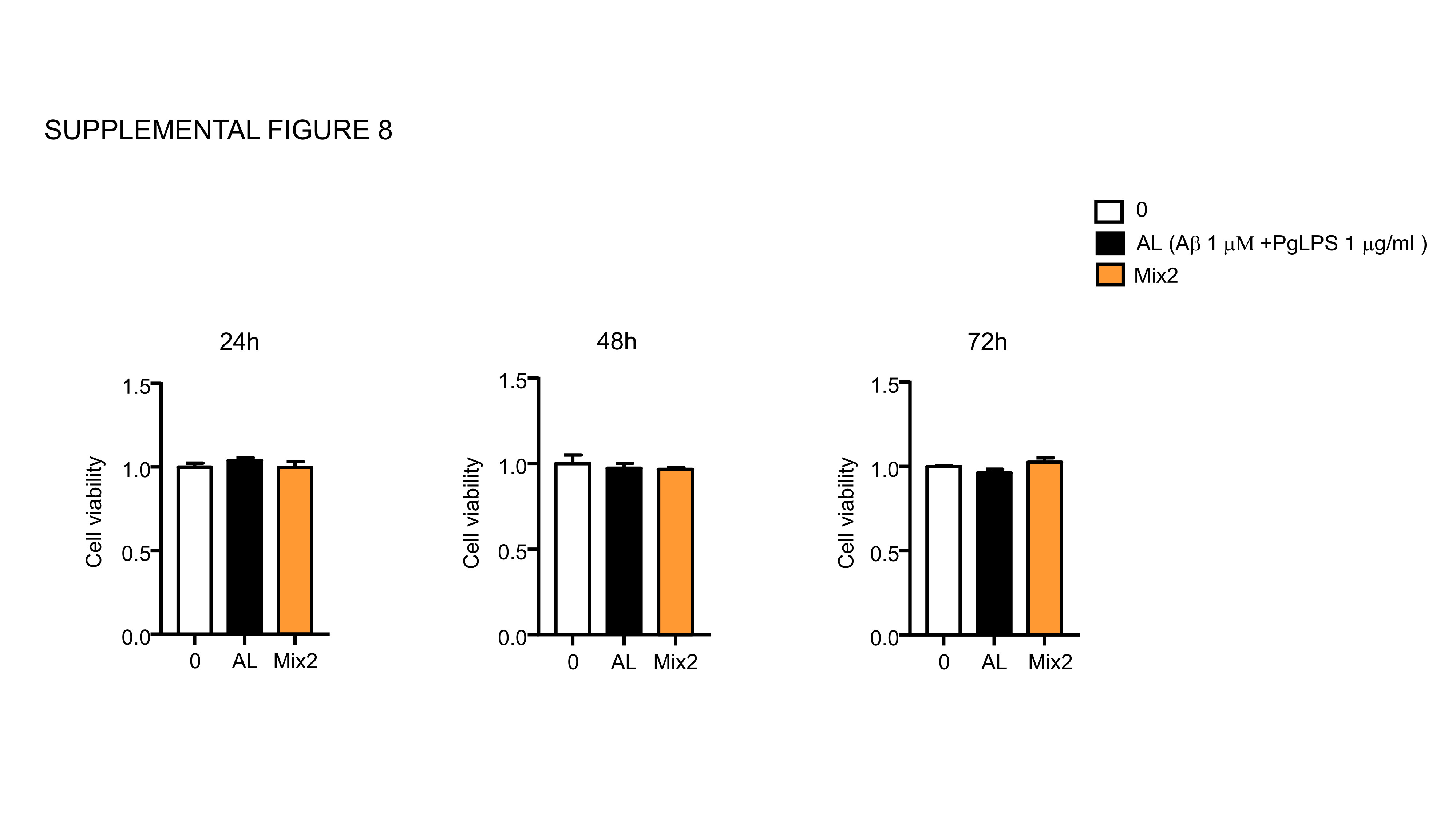

Supplement: Supplementary file 1 — Appendix S1. [file FSN3-12-3745-s001.zip › fsn34045-sup-0009-FigureS8.tif]

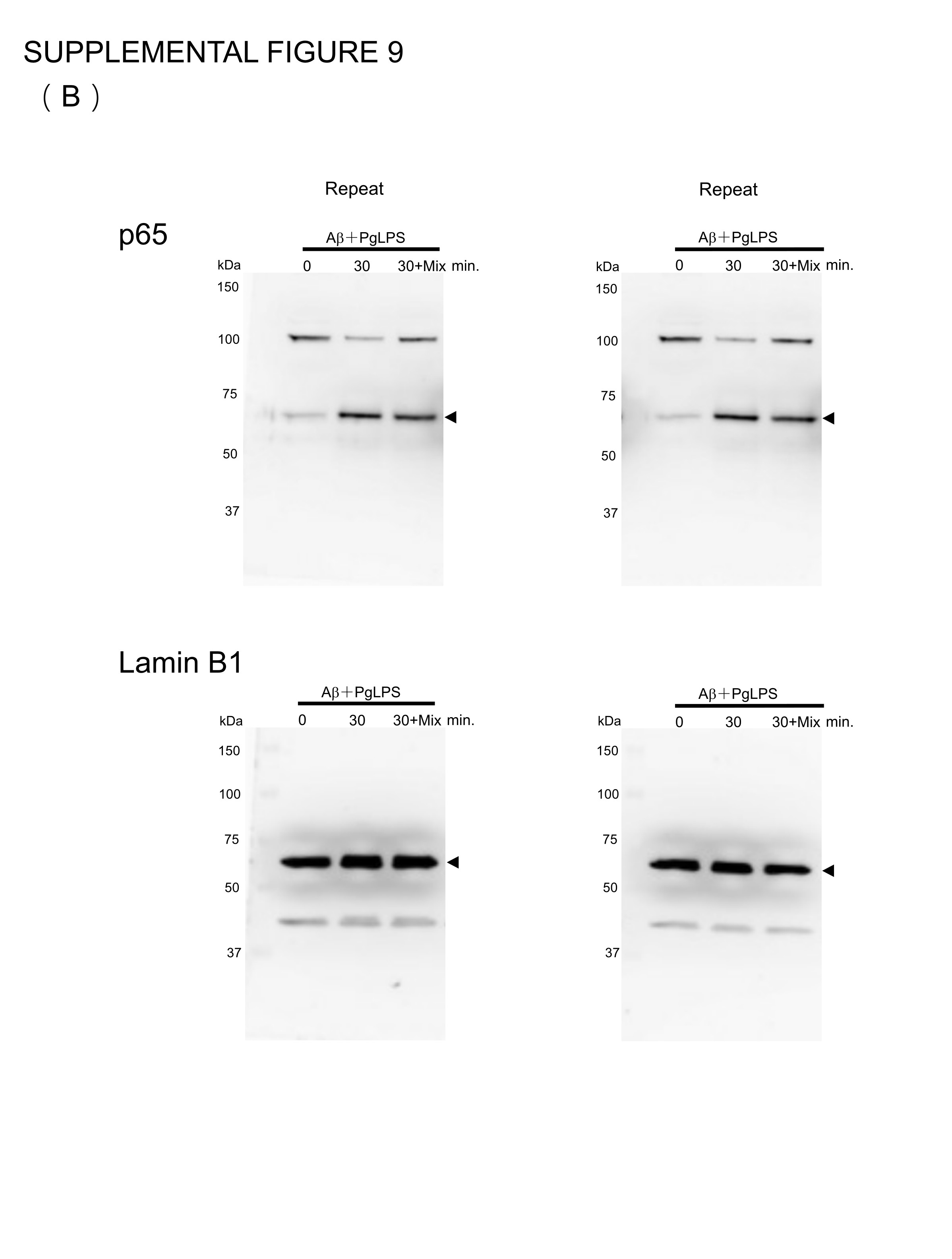

Supplement: Supplementary file 1 — Appendix S1. [file FSN3-12-3745-s001.zip › fsn34045-sup-0010-Figure 9B.tif]

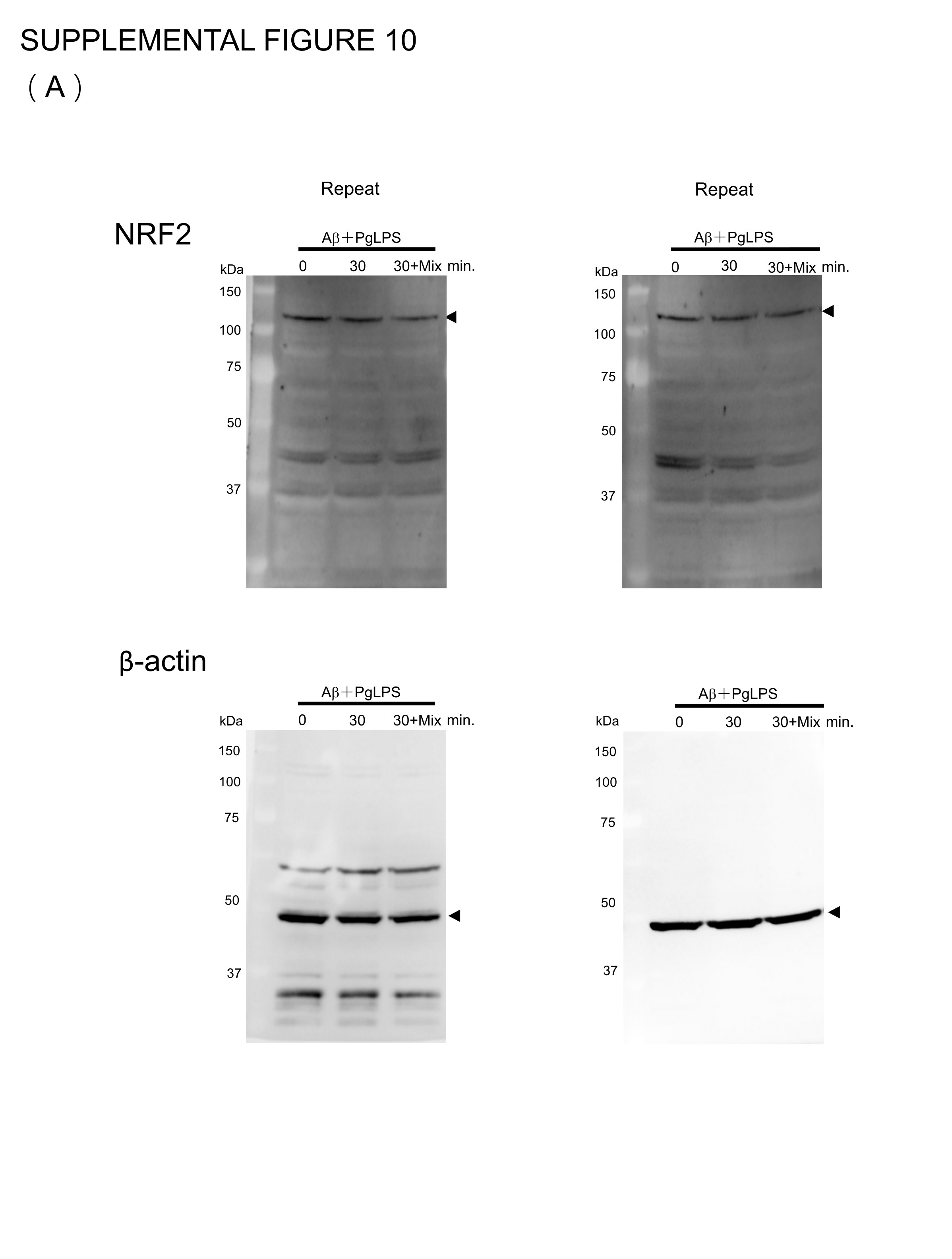

Supplement: Supplementary file 1 — Appendix S1. [file FSN3-12-3745-s001.zip › fsn34045-sup-0011-Figure 10A.tif]

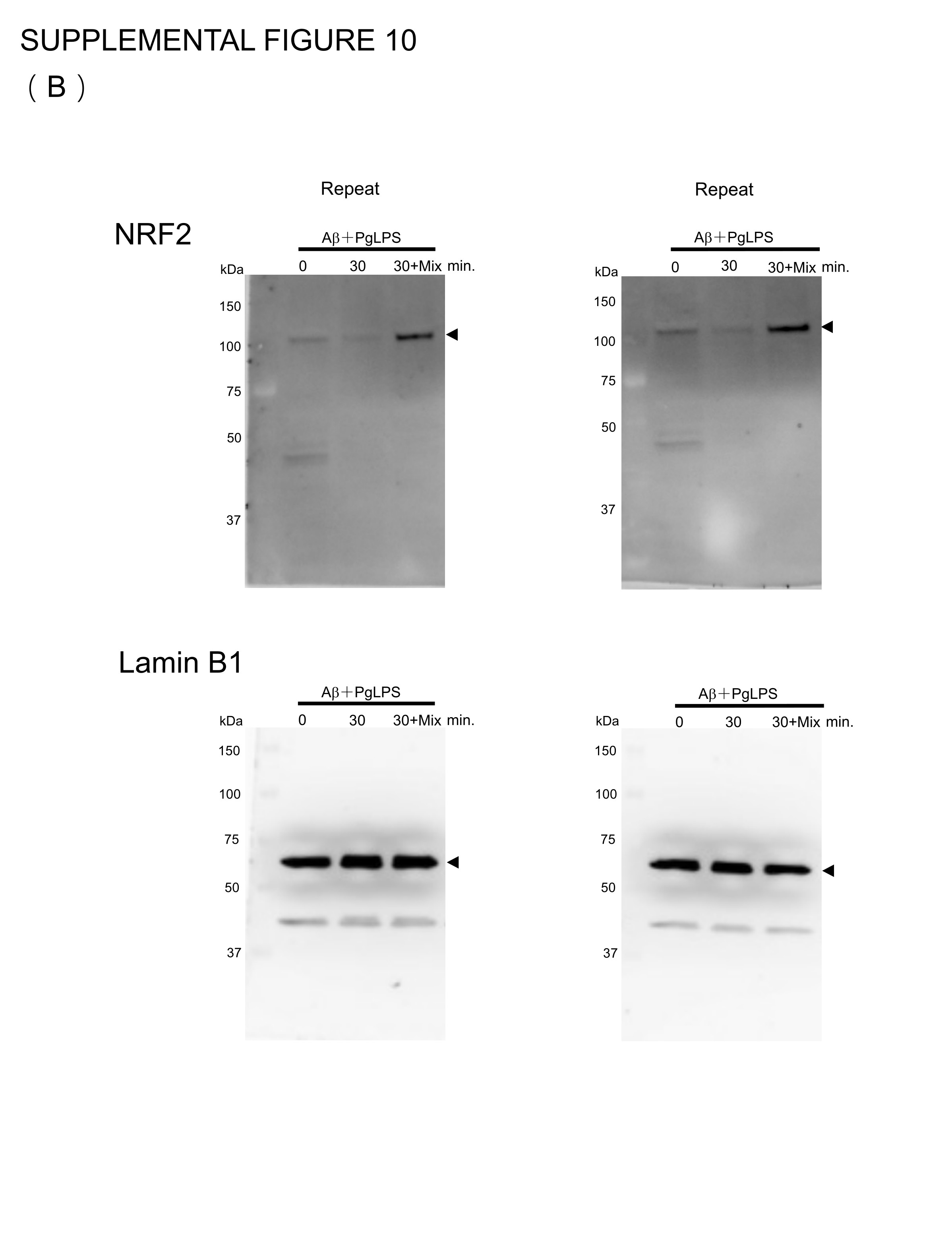

Supplement: Supplementary file 1 — Appendix S1. [file FSN3-12-3745-s001.zip › fsn34045-sup-0012-Figure 10B.tif]
